# Plk4 and Aurora A cooperate in the initiation of acentriolar spindle assembly in mammalian oocytes

**DOI:** 10.1083/jcb.201606077

**Published:** 2017-11-06

**Authors:** Leah Bury, Paula A. Coelho, Angela Simeone, Samantha Ferries, Claire E. Eyers, Patrick A. Eyers, Magdalena Zernicka-Goetz, David M. Glover

**Affiliations:** 1 Department of Genetics, University of Cambridge, Cambridge, England, UK; 2 Wellcome Trust/Cancer Research UK Gurdon Institute, University of Cambridge, Cambridge, England, UK; 3 Department of Physiology, Development, and Neuroscience, University of Cambridge, Cambridge, England, UK; 4 Department of Biochemistry, Institute of Integrative Biology, University of Liverpool, Liverpool, England, UK

## Abstract

Establishing the bipolar spindle in mammalian oocytes after their prolonged arrest is crucial for meiotic fidelity and subsequent development. In contrast to somatic cells, the first meiotic spindle assembles in the absence of centriole-containing centrosomes. Ran-GTP can promote microtubule nucleation near chromatin, but additional unidentified factors are postulated for the activity of multiple acentriolar microtubule organizing centers in the oocyte. We now demonstrate that partially overlapping, nonredundant functions of Aurora A and Plk4 kinases contribute to initiate acentriolar meiosis I spindle formation. Loss of microtubule nucleation after simultaneous chemical inhibition of both kinases can be significantly rescued by drug-resistant Aurora A alone. Drug-resistant Plk4 can enhance Aurora A–mediated rescue, and, accordingly, Plk4 can phosphorylate and potentiate the activity of Aurora A in vitro*.* Both kinases function distinctly from Ran, which amplifies microtubule growth. We conclude that Aurora A and Plk4 are rate-limiting factors contributing to microtubule growth as the acentriolar oocyte resumes meiosis.

## Abstract (precis)

Establishing the spindle in mammalian oocytes after their prolonged arrest occurs in the absence of centrioles and is crucial for meiotic fidelity. Bury et al. show that this requires concerted activity of microtubule organizing center–associated Aurora A and Plk4, which are usually found at centrioles.

## Introduction

Proper spindle assembly is critical for chromosome alignment and segregation during meiotic and mitotic cell divisions. The first meiosis in female mammals is extraordinary, because it arises from a state of prophase arrest that, depending upon the species, can persist for many decades from birth. Defects in spindle formation during this division correlate with chromosome segregation errors and are a leading cause of infertility and embryonic aneuploidy (Hassold and Hunt, 2001). In somatic animal cells and spermatocytes, the pericentriolar material (PCM) component of centrosomes nucleates microtubules that “search and capture” chromosomes as the bipolar spindle forms (Kirschner and Mitchison, 1986a,b). However, spindle assembly can still occur after elimination of functional centrosomes in cultured cells (Khodjakov et al., 2000; Mahoney et al., 2006) or the whole organism (Megraw et al., 2001; Azimzadeh et al., 2012). Centrosomes do, however, enhance mitotic fidelity (Delattre and Gönczy, 2004; Zamora and Marshall, 2005; McCoy et al., 2015). Nevertheless, in most metazoans, centrioles are naturally eliminated during oogenesis before female meiosis (Delattre and Gönczy, 2004) Thus, high fidelity of chromosome transmission during meiosis I in the oocyte, essential to correctly establish the next generation, relies on acentrosomal spindle assembly (Heald et al., 1996).

The small GTPase Ran was the first molecule found to regulate acentrosomal microtubule nucleation. Its role has been most extensively studied in *Xenopus laevis* extracts (Kalab et al., 1999; Ohba et al., 1999; Wilde and Zheng, 1999), where a gradient of GTP-bound Ran around chromatin promotes the release of spindle assembly factors from inhibitory importins (Caudron et al., 2005; Bastiaens et al., 2006; Kaláb et al., 2006; Forbes et al., 2015). Although the Ran-GTP pathway increases microtubule density around chromosomes in mouse oocytes (Schuh and Ellenberg, 2007), neither interfering with Ran-GTP itself in mouse (Dumont et al., 2007; Schuh and Ellenberg, 2007) or *Xenopus* (Dumont et al., 2007) oocytes nor inhibiting certain Ran effectors such as hepatoma up-regulated protein (Breuer et al., 2010), it prevents meiosis I spindle assembly. These observations, strengthened by the finding that enucleated oocytes do not develop any spindle-like structure (Schuh and Ellenberg, 2007), led to the suggestion that alternative factors must promote spindle formation during the resumption of meiosis I after prolonged arrest. Importantly, such limiting factors governing the kinetics of the early stages of microtubule assembly to form a functional meiosis I spindle remain to be identified. This raises a fundamental question: how is spindle formation initiated during the first meiotic division?

A partial explanation is provided by the presence of multiple microtubule organizing centers (MTOCs) in the oocyte cytoplasm (Maro et al., 1985; Messinger and Albertini, 1991; Van Blerkom, 1991; Combelles and Albertini, 2001). Although acentriolar, these MTOCs contain PCM components, including CEP192 (Clift and Schuh, 2015), γ-tubulin (Gueth-Hallonet et al., 1993; Palacios et al., 1993), and pericentrin (Carabatsos et al., 2000). Studies of meiotic maturation in live oocytes (Schuh and Ellenberg, 2007) have revealed that the MTOCs closely surround the nucleus and contribute to an increase in microtubule density at the time of nuclear envelope breakdown (NEBD). However, the regulatory components that enable the initiation of microtubule nucleation and growth after the oocyte’s prolonged arrest in prophase are still unknown.

Here, we have discovered that Plk4 and Aurora A together contribute to trigger rapid growth of microtubules at initial stages of spindle formation in the acentriolar mouse oocyte. Combining chemical genetics with live imaging analysis, we demonstrate an overlapping function of these two kinases in initiating microtubule growth in formation of the meiosis I spindle. Inhibition of either Plk4 or Aurora A kinase alone leads to a diminution of microtubule growth after NEBD in a manner characteristic for each kinase. However, extreme loss of microtubule nucleation results from simultaneous inhibition of both kinases. We present evidence that the two kinases have both independent and interdependent roles in microtubule nucleation. These functions are independent of the Ran pathway, which acts to amplify microtubule growth to promote formation of the acentriolar spindle in the extended prometaphase of this critical first meiotic division.

## Results

### MTOC-associated Plk4 contributes to microtubule nucleation upon resumption of meiosis

**Figure 1.**
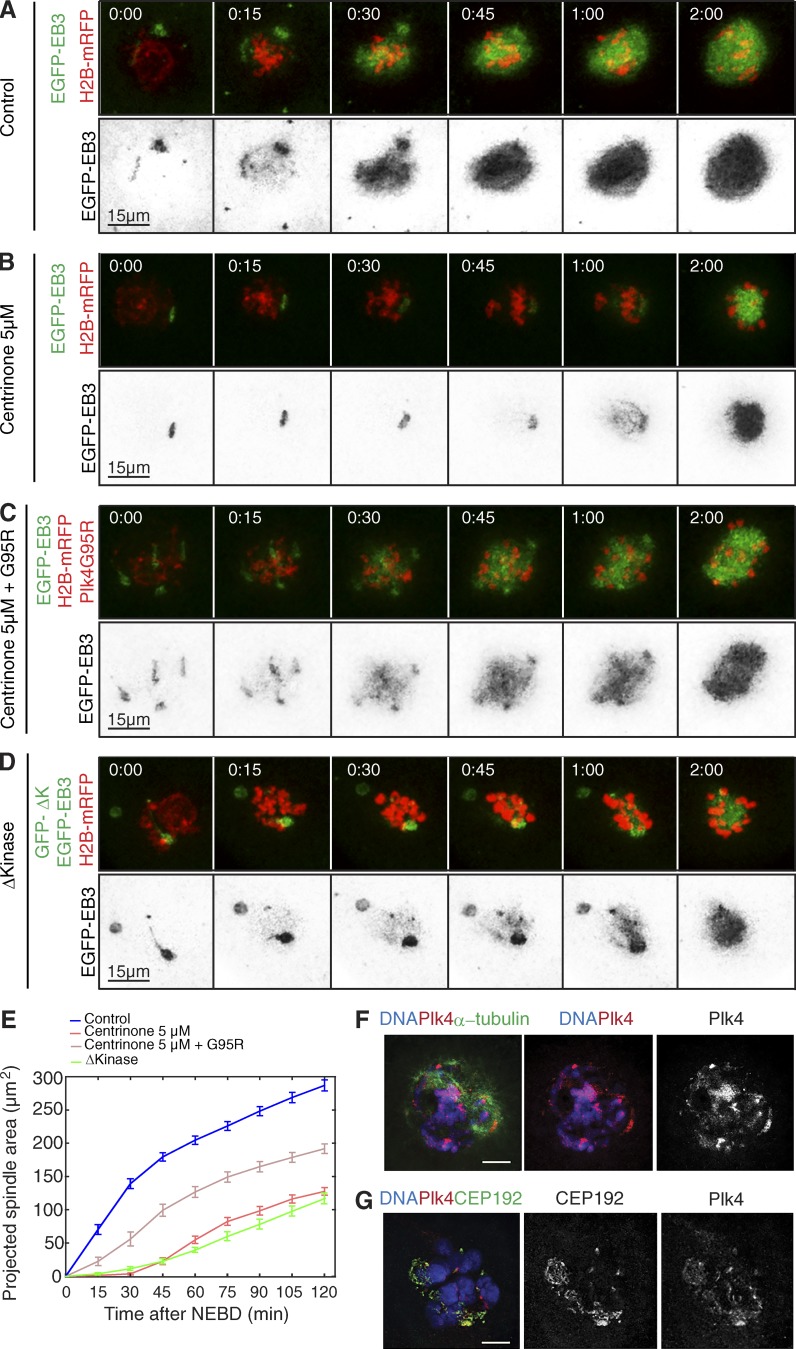
**Plk4 localizes to MTOCs in the mouse oocyte and facilitates microtubule growth upon resumption of meiosis.** (A-E) Time-lapse series of oocytes injected with mRNA encoding EGFP-EB3 (gray, top; inverted, bottom) and histone H2B-mRFP (red, top). Oocytes were imaged as controls (A), under Plk4 inhibition by treatment with 5 µM centrinone (B), after coinjection of mRNA encoding the centrinone-resistant point mutant Plk4G95R the in presence of 5 µM centrinone (C), or while coexpressing Plk4Δkinase (D). Time (in hours:minutes) relative to NEBD. See Video 1. (E) Quantification of the size of the projected spindle area. Plk4 inhibition by expression of the dominant-negative Plk4Δkinase (GFP-ΔK; *n* = 13) or by treatment with centrinone at 5 µM (*n* = 26). Centrinone significantly reduces the kinetics of microtubule growth, as determined by the size of the spindle area, compared with controls (*n* = 44). Defects caused by centrinone treatment are significantly rescued by coexpression of Plk4G95R (*n* = 25). Error bars indicate SEM. P-values are shown in Table S1. See also Video 1. (F and G) Localization of Plk4 at MTOCs, revealed by immunostaining of oocytes with anti-Plk4, anti-CEP192, and anti–α-tubulin antibodies, in the indicated colors. DNA is shown in blue. Bars, 10 µm.

In considering which factors might promote microtubule nucleation in the oocyte upon resumption of meiosis, we first asked whether they might be similar to those required for spindle formation from acentriolar MTOCs in the early mouse embryo (Coelho et al., 2013). It was shown in the embryo that this requires Plk4, a protein kinase more widely known for its roles in centriole duplication (Bettencourt-Dias et al., 2005; Habedanck et al., 2005). To determine whether Plk4 might also have a similar role in the acentriolar mouse oocyte, we first chose to treat oocytes with centrinone, a potent and selective small-molecule inhibitor of Plk4 (Wong et al., 2015). To visualize the dynamics of growing microtubules and chromosomes, we coinjected germinal vesicle–stage oocytes with mRNAs for EGFP-EB3 and histone H2B-mRFP and measured the increase in size of the projected spindle area with time. Zero time (00:00) was established as the time when EB3-GFP signal was first observed inside the nucleus, indicating NEBD. In control oocytes, we observed microtubule growth from MTOCs surrounding the condensed chromosomes, as previously described (Schuh and Ellenberg, 2007), leading to assembly of a robust spindle after NEBD (zero time). After centrinone treatment, bipolar spindles still formed but with a strikingly slower kinetics. Because we observed a delay in the initial nucleation of microtubules, we quantified the projected spindle area over an interval of 2 h after NEBD during which control oocytes reach 80% of the plateau level. To compare the effects of inhibiting Plk4 function, we noted both the extent of microtubule nucleation within this time period and the time taken for the spindle to reach 50% of its maximal expansion within this 2-h interval (t50^2h^; see Materials and methods). We found that the spindles of centrinone-treated oocytes developed to 51% of the size of control spindles by 2 h and required 55.4 ± 3.8 min to reach 50% of their size at 2 h (Fig. 1, A, B, and E; Table S2; and Video 1).

These observations suggested that Plk4 is required for efficient nucleation of microtubules in the acentriolar oocyte just as it is in the early embryo (Coelho et al., 2013). However, in contrast to the early embryo, where Plk4 depletion or expression of dominant-negative Plk4 results in monopolar spindles, bipolar spindles still formed in centrinone-treated oocytes. To ensure that this was not a peculiarity of the pharmacological inhibition of Plk4, we treated embryos with centrinone and monitored spindle formation. This resulted in monopolar spindle formation (Fig. S1), recapitulating the phenotype observed previously (Coelho et al., 2013). Thus, the reduced kinetics of microtubule nucleation we observe in centrinone-treated oocytes is likely to result from Plk4 inhibition.

Notwithstanding this finding, a difficulty in using pharmacological approaches to inhibit protein kinases is that, to our knowledge, without exception, no small-molecule inhibitor is completely specific for a single target. Thus, to validate the target, we created a drug-resistant allele of murine Plk4 (Plk4 G95R), which, based on previous studies with human PLK4 (Scutt et al., 2009; Sloane et al., 2010), spares catalytic activity and is resistant to various “type I” ATP-competitive protein kinase inhibitors. The drug-resistant Plk4 variant was 67.4% effective at rescuing the effects of centrinone treatment at the very early stages of microtubule nucleation after NEBD in mouse oocytes (Fig. 1, C and E; and Video 1). That this mutant did not fully rescue in vivo could be explained if centrinone possesses another (unknown) target in oocytes that is required for microtubule growth. Moreover, we cannot rule out that G95R Plk4 might possess a subtly different catalytic activity or substrate specificity when expressed in cells, although we consider this unlikely. Nevertheless, the 67.4% rescue achieved by Plk4 G95R indicates a substantial involvement of Plk4 in the initiation of microtubule nucleation.

To determine whether the effect of centrinone could be attributed to Plk4 inhibition, we used the alternative method of microinjecting germinal vesicle–stage oocytes with synthetic mRNA encoding Plk4Δkinase. This Plk4 variant was previously shown to exhibit a dominant-negative effect upon microtubule nucleation at mouse embryo acentriolar MTOCs (Coelho et al., 2013). We found that in oocytes expressing Plk4Δkinase, the projected spindle area reached only 40.8% of control at 2 h and spindles assembled with slower kinetics (t50^2h^ = 74 ± 4.2 min compared with 38.7 ± 7.8 min in controls; Fig. 1, A, D, and E; Table S2; and Video 1). This effect is extremely similar to that of centrinone treatment.

These observations led us to consider whether Plk4 might be associated with the MTOCs, which are known to contain several molecules associated with PCM of conventional centrosomes (Gueth-Hallonet et al., 1993; Palacios et al., 1993; Carabatsos et al., 2000). In accord with this notion and in agreement with the finding of Plk4 at the acentriolar MTOCs of early mouse embryos (Coelho et al., 2013), we found that Plk4 was enriched near condensing chromosomes and on cytoplasmic MTOCs in the vicinity of the centrosomal protein Cep192 (Fig. 1, F and G).

**Figure 2.**
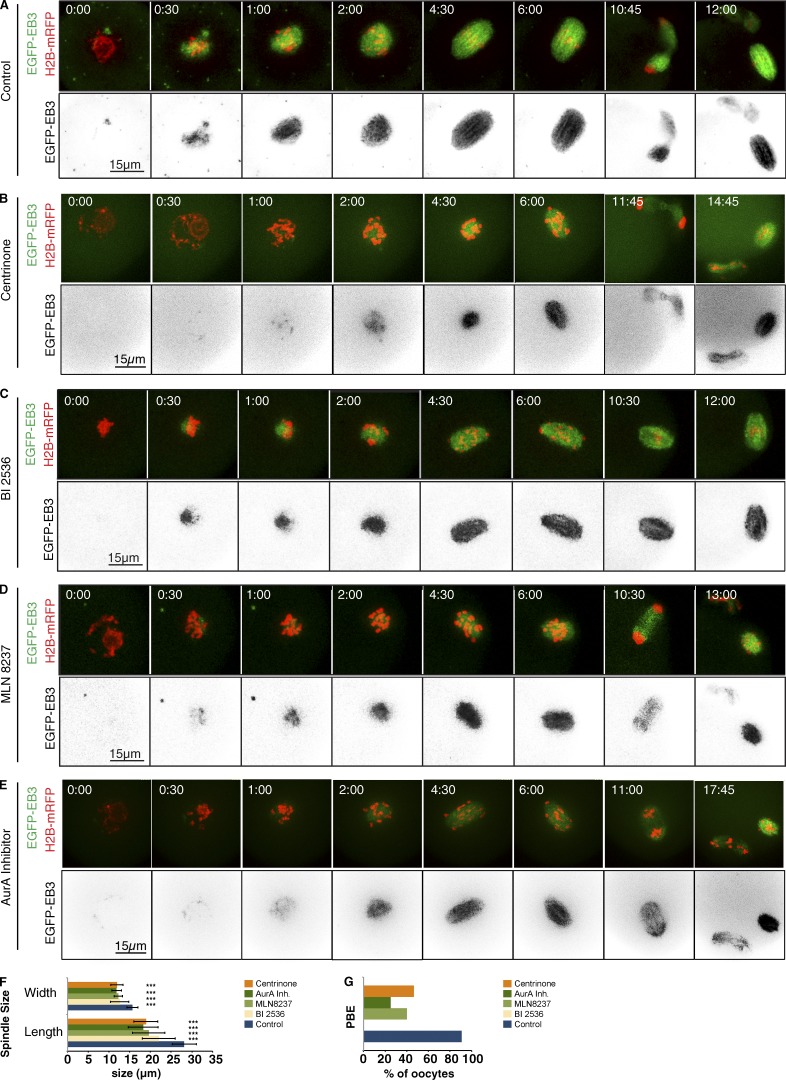
**Aurora A, Plk4, or Plk1 inhibition differently affect meiosis I progression.** Time-lapse series of mouse oocytes expressing EGFP-EB3 (green, top; gray, bottom) and histone H2B-mRFP (red, top). Depicted are controls (A), oocytes treated with 2 µM centrinone (B), oocytes under Plk1 inhibition by treatment with 100 nM of the Plk1 inhibitor BI2536 (C), and oocytes treated with 1 µM Aurora A inhibitor 1 (AurA Inh 1) and 500 nM MLN8237 (E). Time in hours:minutes relative to NEBD. (A–E) Representative still images from NEBD until establishment of spindle bipolarity, anaphase I onset, and polar body extrusion under the different experimental conditions. After Aurora A inhibition by Aurora A inhibitor 1 (*n* = 21) or MLN8237 (*n* = 15), oocytes show a delay in establishment of a bipolar spindle compared with controls (*n* = 20), and they can undergo anaphase I without proper bivalent congression to the metaphase I plate. Under Plk4 inhibition by centrinone (*n* = 10), oocytes eventually form a bipolar spindle. Similarly, as a result of Plk1 inhibition using BI2536 (*n* = 15), oocytes exhibit a delay in establishing a metaphase plate, and anaphase onset is not observed within 17 h. (F) Prometaphase spindle length and width in controls (*n* = 23) compared with oocytes after inhibition of Plk1 (BI2536, *n* = 19), Plk4 (centrinone, *n* = 23), or Aurora A (Aurora A inhibitor 1, *n* = 22; and MLN8237, *n* = 11). Inhibition of each of the three kinases significantly decreases both prometaphase spindle length and width (***, P < 0.001). Error bars indicate SD of the mean. (G) Percentage of polar body extrusion (PBE) in control oocytes as well as after inhibition of Plk1 (BI2536, *n* = 19), Plk4 (centrinone, *n* = 23), or Aurora A (Aurora A inhibitor 1, *n* = 22; and MLN8237, *n* = 11). Inhibition of Aurora A or Plk4 decreases the efficiency of polar body extrusion over 17 h of imaging. Inhibition of Plk1 completely prevents polar body extrusion within this time period.

Collectively, these experiments indicate that Plk4 is associated with MTOCs where it participates in triggering microtubule nucleation after the prolonged state of premeiotic prophase arrest, just as it does in the early embryo. However, in contrast to the early embryo, a bipolar spindle did eventually form in the oocyte in the absence of Plk4 function (Fig. 2). Thus, other factors are able to support sufficient microtubule nucleation to enable spindle formation in the absence of Plk4, and this is reflected in residual microtubule nucleation that we still see when Plk4 is inhibited in the oocyte.

### Aurora A also contributes to the initiation of microtubule nucleation

In considering whether other protein kinases might promote microtubule nucleation in the oocyte, we investigated potential roles of Aurora A and Plk1, because these centrosome-associated kinases possess this function in the PCM in mitosis (Marumoto et al., 2005; Barr and Gergely, 2007; Archambault and Glover, 2009). To this end, we treated mouse oocytes with target-validated potent small-molecule inhibitors of Aurora A (Aurora A inhibitor 1 [Aliagas-Martin et al., 2009] and MLN8237 [Sloane et al., 2010]) or Plk1 (BI2536; Steegmaier et al., 2007; Scutt et al., 2009).

**Figure 3.**
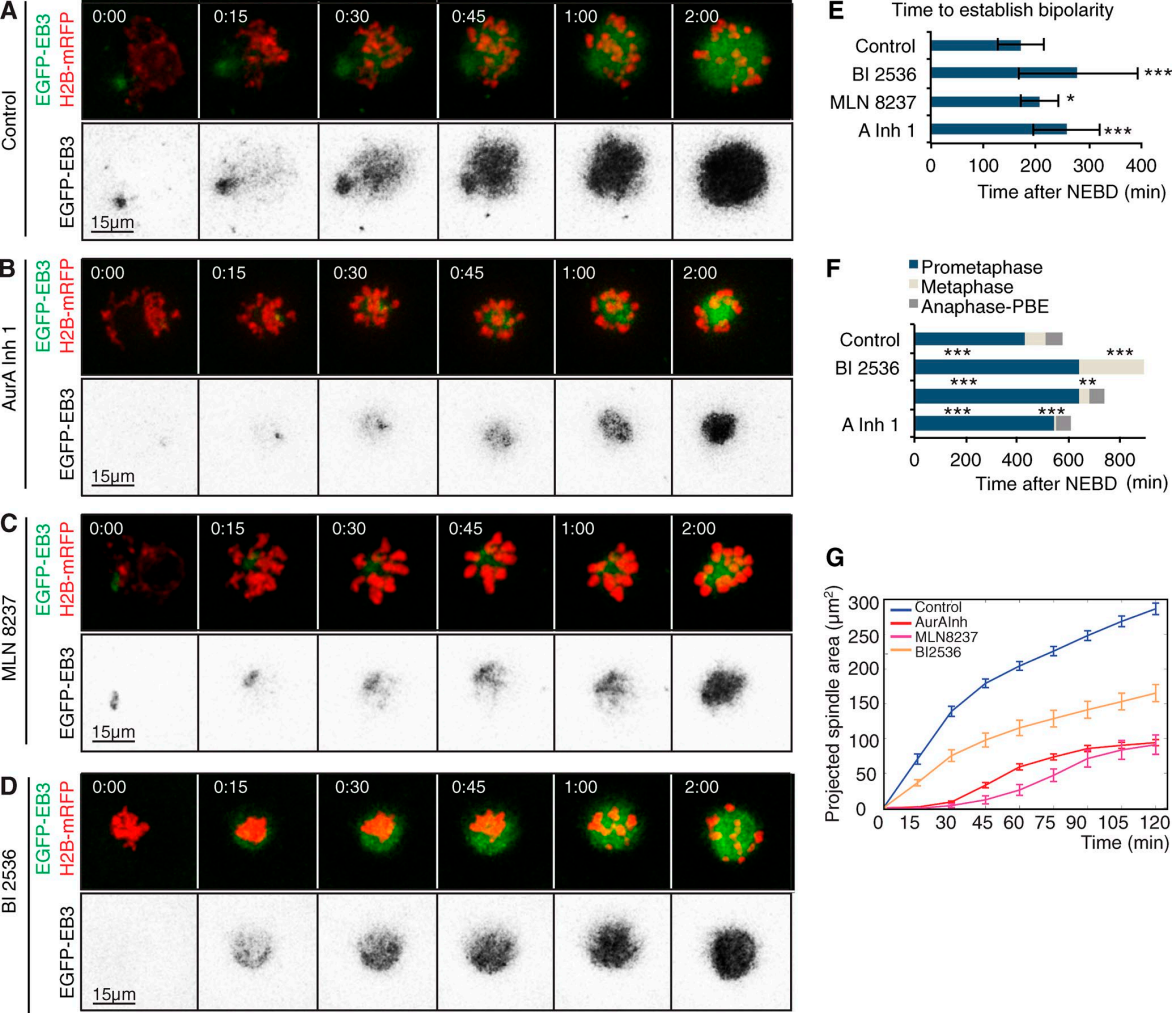
**Aurora A, but not Plk1, is required for microtubule growth upon NEBD during mouse oocyte meiosis I.** (A–D) Time-lapse series of mouse oocytes expressing EGFP-EB3 (green, top; gray, bottom) and histone H2B-mRFP (red, top). Depicted are controls (A), oocytes treated with 1 µM Aurora A inhibitor 1 (AurA Inh 1; B) or 500 nM MLN8237 (C), and under Plk1 inhibition by treatment with 100 nM of the Plk1 inhibitor BI2536 (D). Time in hours:minutes relative to NEBD. (E) Quantification of the duration from NEBD to establishment of spindle bipolarity. Plk1 inhibition (BI2536) and Aurora A inhibition by Aurora A inhibitor 1 or MLN8237 significantly prolongs the time until establishment of a bipolar spindle (***, P < 0.001; *, P < 0.05) compared with controls (*n* = 20). Error bars indicate SD of mean. (F) Time from NEBD to establishment of a metaphase I plate until polar body extrusion (PBE). Plk1 and Aurora A inhibition significantly prolongs the time spent in metaphase. Additionally, Plk1 inhibition causes an arrest at metaphase I. The time spent in metaphase is significantly shorter for oocytes treated with Aurora A inhibitor 1 or MLN8237 than for controls (***, P < 0.001; **, P < 0.01; *, P < 0.05). Error bars indicate SD of mean. (G) Measurement of the size of projected spindle area throughout time. Inhibition of Aurora A (AurA Inh 1, *n* = 21; MLN8237, *n* = 5) or Plk1 (BI2536, *n* = 15) significantly reduces the spindle size reached at 2 h; however, the kinetics of microtubule growth is only affected by inhibition of Aurora A. For p-values and t50^2h^, see Tables S1 and S3, respectively. Time 00:00 corresponds to time of NEBD and is determined by invasion of the EGFP-EB3 signal within the area previously occupied by the nucleus. Error bars indicate SEM. See also Video 2.

We found that treatment of oocytes with either Aurora A inhibitor 1 or MLN8237 (Fig. 2, D–F; Fig. 3, A–C; Fig. S2; Table S3; and Video 2) significantly reduced the final spindle area to 32.7% or 36% of controls, respectively (Fig. 3 G). Treatment with either Aurora A inhibitor 1 or MLN8237 treatment also slowed the kinetics of spindle expansion, reflected in an increased t50^2h^ from 38.7 ± 7.8 min in controls to 55 ± 2.8 or 72.77 ± 1.4 min after the respective treatments (Table S2). This largely reflected a lag in the initiation of microtubule nucleation (Fig. 3, B, C, and G). Expression of a drug-resistant Aurora A variant restored 76% of microtubule growth in a 2-h period after Aurora A inhibitor I treatment (G229L; Fig. S2 D and Video 2). When we followed the effects of Aurora A inhibition over an extended interval of up to 18 h, we found that the time taken to establish a bipolar spindle (Fig. 2, D and E; and Fig. 3 E) and the time spent in prometaphase (Fig. 3 F) were both significantly increased. Metaphase (judged visually by the alignment of chromosomes at the spindle’s equatorial zone; see also Materials and methods) was eventually attained, and oocytes then undertook the first meiotic division (Fig. 2 and Fig. 3, E and F).

In contrast to the effects of Aurora A inhibition, there was no lag in the onset of spindle microtubule growth in BI2536-treated versus control oocytes, and the time taken for microtubules to grow to 50% of their levels at 2 h was much less affected (t50^2h^ = 41.1 ± 7.3 min; Fig. 3, D and G; and Table S2). Microtubule density was diminished 2 h after Plk1 inhibition to 56.0% of control levels. However, the effect on microtubule density was not as dramatic as after inhibition of either Plk4 or Aurora A (Figs. 2 F and 3 G). This is in accord with the observations of Clift and Schuh (2015), who found that inhibition of Plk1 did not significantly reduce the amount of microtubules at MTOCs, although it did prevent MTOC fragmentation into multiple microtubule organizing bodies. Our observations of a delay in establishing spindle bipolarity with an extended prometaphase culminating in arrest in a metaphase-like state (Fig. 2, C and G; Fig. 3, D–F; and Table S3) are also consistent with these previous observations (Clift and Schuh, 2015).

**Figure 4.**
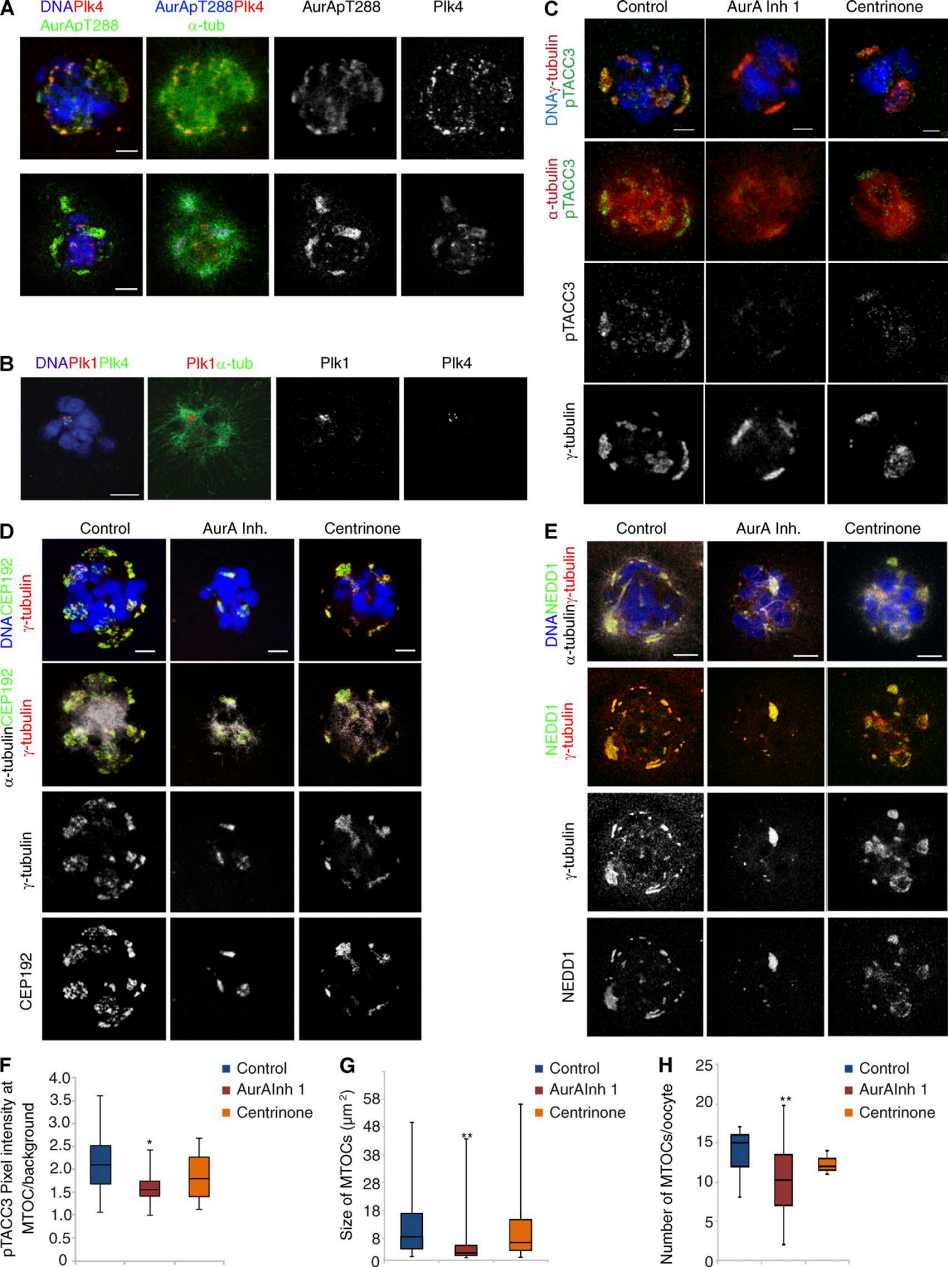
**Aurora A activity is required for the correct size and number of MTOCs in oocytes.** (A) Localization of Plk4 and Aurora A phosphorylated at threonine residue 288 (AurApT288) revealed by immunostaining of oocytes with anti–Plk4 (red), anti–α-tubulin (green), and anti–AurApT288 (red) antibodies. Separated channels in gray are also shown as indicated; DNA is shown in blue. Both kinases colocalize at MTOCs. (B) Localization of PlK1 at MTOCs revealed by immunostaining of meiosis I oocytes with anti-Plk1 (red) and anti–α-tubulin (green). Plk1 and Plk4 channels are also shown in monochrome and DNA in blue. (C) Meiosis I oocytes stained to visualize DNA (blue), γ-tubulin (red), and phospho-TACC3 (pTACC3, green; top) or α-tubulin (red, middle) and pTACC3 (monochrome, bottom). Depicted are controls compared with conditions in which Aurora A and Plk4 were inhibited by treatment with Aurora A inhibitor 1 or centrinone, respectively. Aurora A inhibition causes a significant reduction of pTACC3 at MTOCs (see bottom panel, pTACC3 in monochrome). (D) Oocytes stained with antibodies against α−tubulin (gray, bottom), DNA (blue, top), γ-tubulin (red or monochrome), and CEP192 (green or monochrome, bottom; A) or stained to visualize α−tubulin (gray, top), DNA (blue, top), γ-tubulin (red or monochrome), and NEDD1 (green or monochrome, bottom; E). Shown are representative images of controls and of oocytes treated with Aurora A inhibitor 1 and centrinone. Bars, 10 µm. (F) Measurement of pTACC3 pixel intensity at the MTOCs relative to the background (*, P < 0.05). Error bars represent SD of the mean. (G and H) Quantifications of the size (G) and number (H) of γ-tubulin–positive MTOCs. Aurora A inhibition, but not treatment of oocytes with the Plk4 inhibitor centrinone, causes a significant reduction in size and number of MTOCs (**, P < 0.01).

Together, these data lead us to suggest that both Plk1 and Aurora A contribute to the initiation of bipolar meiosis I spindle formation but that there is a greater requirement for Aurora A than Plk1 to promote timely onset of microtubule nucleation at NEBD. This, alongside our observations of diminished microtubule growth upon down-regulating Plk4, suggests that Plk4 and Aurora A cooperate to initiate microtubule nucleation, leading us to evaluate whether these kinases were associated with MTOCs. To determine whether the area of the spindle illuminated by EGFP-labeled EB3 in these experiments reflects the distribution of total tubulin, we performed immunostaining of fixed preparations of oocytes to reveal α-tubulin after chemical inhibition of Aurora A or Plk4 kinases. This confirmed a comparable effect of the compounds on total α-tubulin levels thus validating spindle volume of EB3 fluorescence as an indicator of spindle size (Fig. S3). Consistent with previous findings (Saskova et al., 2008; Ding et al., 2011; Solc et al., 2012), we detected Aurora A at the MTOCs of mouse oocytes in its active phosphorylated form (pT288 Aurora A; Fig. 4 A). Additionally, Plk4 and Plk1 were also present at these MTOCs in meiosis I (Fig. 4, A and B; Solc et al., 2015). To assess how this reflects spatial activity of Aurora A, we examined the phosphorylation state of TACC3, an established substrate of Aurora A, which associates with the minus ends of microtubules on centrosomes (Barros et al., 2005; Kinoshita et al., 2005). Consistent with the localization of active (pT288) Aurora A on the MTOCs (Tyler et al., 2007) and in agreement with a previous study (Brunet et al., 2008), we detected pTACC3 at MTOCs, visualized with γ-tubulin. Importantly, the intensity of the pTACC3 signal was significantly reduced as a result of Aurora A inhibition (Fig. 4, C and F), but we were still able to detect pTACC3 at MTOCs after Plk4 inhibition, with a similar distribution as in control oocytes (Fig. 4, C and F). This suggests that Plk4 can regulate microtubule growth through pathways that do not involve Aurora A–mediated phosphorylation of TACC3. It seemed from these observations that the numbers of foci of pTACC3 on microtubules were reduced significantly after the inhibition of Aurora A kinase, but not Plk4. To determine the effects of inhibiting either kinase upon cytoplasmic MTOCs, we treated oocytes with Aurora A or Plk4 inhibitors and stained them to reveal the centrosome components γ-tubulin, a component of the minus end–associated γ-tubulin ring complex; the γ-tubulin partner NEDD1; or the PCM-associated, Plk4 partner Cep192. Together, this revealed that inhibition of Aurora A, but not Plk4, led to a significant reduction in size and number of MTOCs (Fig. 4, D, E, G, and H). Thus, both Plk4 and Aurora A appear at MTOCs and in the vicinity of chromosomes, and at least some of their roles in initiating microtubule growth are independent of one another, with Aurora A being required for the correct distribution of MTOCs.

### The requirement for Aurora A predominates over Plk4 to initiate microtubule nucleation

**Figure 5.**
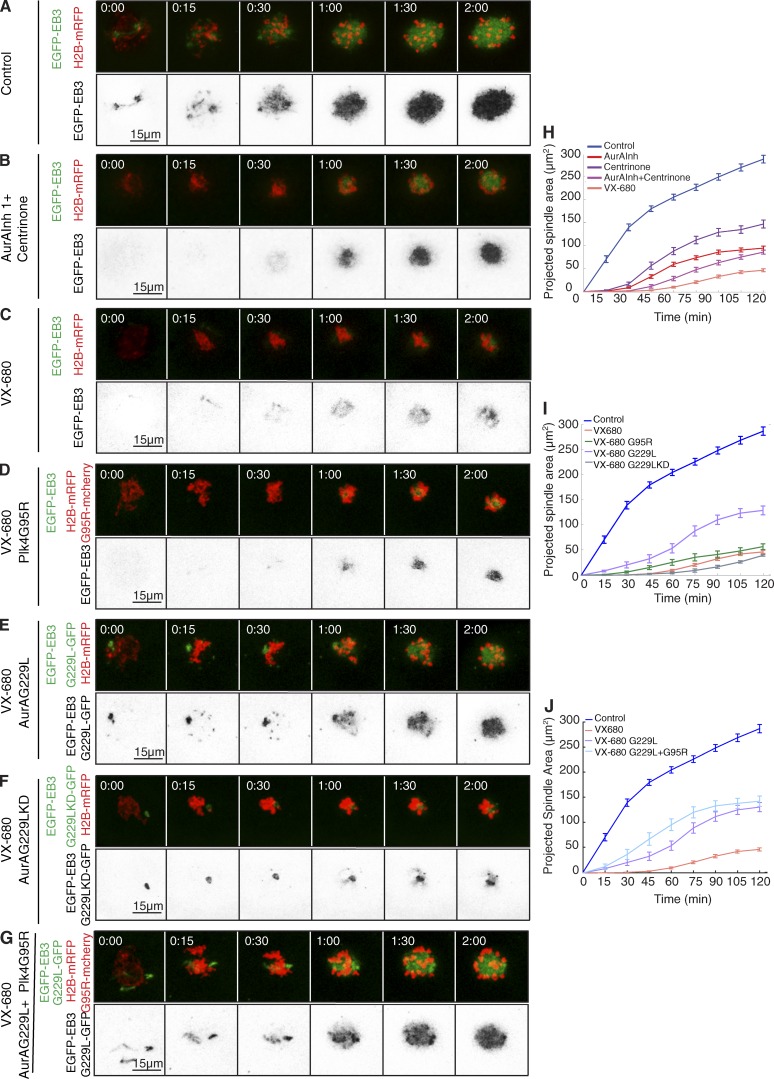
**Requirement for Aurora A predominates over Plk4 to initiate microtubule nucleation.** (A–G) Time-lapse series of oocytes injected with mRNA encoding EGFP-EB3 (green, top; inverted, bottom) and H2B-mRFP (red, top). Progression through meiosis I was followed in controls (A) and under simultaneous inhibition of Aurora A and Plk4 by a combination of Aurora A inhibitor 1 and centrinone (B) and treatment with 1 µM VX-680 (C). To assess reversion of the defects in microtubule growth caused by VX-680, oocytes were coinjected with mRNAs for the Plk4 point mutant G95R (D), Aurora A G229L (E), kinase-dead Aurora A G229 (F), and both Aurora A G229L and Plk4 G95R (G). (H–J) Quantifications of the size of the projected spindle area over time. (H) Compared with single kinase inhibition (AurA Inh1, *n* = 33; centrinone, *n* = 22), treatment with Aurora A inhibitor 1 and centrinone in combination (AurA Inh1 + centrinone, *n* = 10) and VX-680 (*n* = 26) further depresses microtubule growth. (I) Defects caused by VX-680 are not restored by kinase-dead Aurora A G229L (*n* = 9), partially rescued by expression of Plk4 G95R (*n* = 10), and significantly restored by expression of inhibitor-resistant Aurora A G229L (*n* = 15). (J) Rescue by Aurora A G229L is enhanced when Plk4 G95R is coexpressed (*n* = 14). Error bars indicate SEM (***, P < 0.001; **P < 0.01; *P < 0.05). See also Tables S1 and S2 and Video 3.

We next addressed the possibility for potential overlap in function of the two kinases by inhibiting both enzymes simultaneously and then determining the extent to which microtubule nucleation could be rescued by drug-resistant variants of either kinase alone or in combination. We could simultaneously inhibit both kinases by treating oocytes with either a combination of Aurora A inhibitor 1 and centrinone (Fig. 5, B and H) or with the single inhibitory agent VX-680 (Fig. 5, C and H; Fig. S4; Table S3; and Video 3). VX-680 potently inhibits both Aurora A and Plk4 with nanomolar affinity because of conservation of amino acids that create the drug-binding site in the respective ATP-binding pockets (Scutt et al., 2009). In both cases, we observed an enhanced inhibitory effect over the inhibition of individual kinases (combined Aurora A Inhibitor 1 and centrinone, spindle size at 2 h: 29.8% of control; t50^2h^ = 72.7 ± 3.4 min; VX-680, spindle size at 2 h: 16.1% of control; t50^2h^ = 78.1 ± 1.7 min; Table S2). However, VX-680 is also known to inhibit Aurora B and C kinases, which are highly similar in their ATP-binding sites (Tyler et al., 2007), resulting in a failure of cytokinesis and polar body extrusion (Yang et al., 2010). Despite the dramatic initial depression of microtubule nucleation by VX-680, it proved possible to rescue progression through mitosis and cytokinesis phenotype resulting from Aurora B/C inhibition in 85% of VX-680–treated oocytes by expressing a VX-680–resistant mutant form of Aurora B (glycine 165 mutated to leucine, the murine equivalent of the human drug-resistant G160L mutant; Scutt et al., 2009; Fig. S4, H, I, and K–M). However, the Aurora B G165L mutant did not rescue the VX-680–mediated defects in microtubule growth around chromatin in the first hour after NEBD, strongly suggesting that these events must arise as a result of the inhibition of kinases other than Aurora B (Fig. S4, I and K–M). Because Aurora C is so similar to Aurora B in terms of drug-binding site, sensitivity to inhibitors, subcellular localization, and functions in late M phase and cytokinesis, it is unlikely to be involved in these early events. VX-680 is also a known inhibitor of several tyrosine kinases (Scutt et al., 2009). Therefore, we tested the consequences of treating oocytes with saracatinib (Chang et al., 2008), which interferes with the activity of Src kinase and a similar group of tyrosine kinases that are also sensitive to VX-680. We found that the initial nucleation of microtubules after saracatinib treatment was indistinguishable from controls, indicating that saracatinib-sensitive tyrosine kinases do not contribute to this process (Fig. S5, F, G, and J). Thus, the dual inhibition of Aurora A and Plk4 resulting from VX-680 treatment is likely to contribute significantly the delay in microtubule growth after NEBD.

To determine whether Plk4 and Aurora A show any interdependency for the initiation of microtubule growth after resumption of meiosis, we inhibited both kinases simultaneously using VX-680 (Fig. 5 C) and then exploited chemical genetics to investigate whether individual drug-resistant alleles of either kinase could overcome the inhibition when expressed exogenously in oocytes. Activity-sparing VX-680–resistant point mutants in the ATP-binding pocket of Aurora A have been previously described (Scutt et al., 2009; Sloane et al., 2010). We found that expression of the mouse counterpart of inhibitor resistant Aurora A (G229L) in VX-680–treated oocytes was sufficient to restore 44.9% of spindle volume 2 h post-NEBD (compared with 16.1% of controls after VX-680 treatment alone; Fig. 5, E and I). Expression of drug-resistant Aurora A also greatly restored the time to attain half the level of microtubule nucleation (t50^2h^ of spindle formation of 63 ± 2.5 min compared with 78.1 ± 1.7 min after VX-680 treatment alone; Fig. 5, E and I; Table S2; and Video 3). In contrast, a kinase-dead variant of Aurora A G229L was unable to restore microtubule growth and indeed had a dominant-negative effect (t50^2h^ = 93.8 ± 7.4 min; Fig. 5, F and I; and Table S2), confirming a requirement for Aurora A catalytic activity for rescue. Expression of an inhibitor-resistant Plk4 allele (G95R) in VX-680–treated oocytes gave little rescue of the delay in microtubule growth (t50^2h^ = 66.6 ± 4.9 min; Fig. 5, D and I; and Table S2) and was unable to restore spindle size (Fig. 5, D and I). However, coexpression of both Plk4 G95R and Aurora A G229L enhanced the rescue of spindle size to 55%, which is beyond that given by expression of drug-resistant enzyme Aurora A G229L alone (Fig. 5, G and J; and Video 3). Their expression also further reduced the time for attaining 50% of spindle size at 2 h (t50^2h^ = 47.9 ± 2 min; Fig. 5, G and J; and Table S2). Thus, Plk4 appears able to influence the kinetics of the initial microtubule nucleation within limits set by Aurora A activity. The inability to achieve complete rescue of VX-680 inhibition with these mutant kinases may reflect an inherent limitation of the function of these mutant enzymes in vivo, or it could indicate the involvement of another kinase in microtubule nucleation that is inhibited by VX-680. Nevertheless, our experiments do point to a substantial involvement of Aurora A in initiating spindle formation. Moreover, the ability of drug-resistant Plk4 to enhance the rescue of VX-680 inhibition by drug-resistant Aurora A while being poorly able to do so alone suggests a set of Plk4 functions that are dependent on Aurora A.

These findings led us to ask whether Plk4’s role in microtubule nucleation might be dependent on Aurora A. To address this, we investigated whether Aurora A or Plk4 could phosphorylate or regulate each other’s catalytic activity in vitro using the highly conserved human Plk4 and Aurora A kinases that (like their mouse counterparts) become resistant to VX-680 when they carry G95R (Plk4) and G216L (Aurora A) substitutions (Fig. S5 A). Although Aurora A was unable to phosphorylate Plk4 (Fig. S5, B and F–K), Plk4 could phosphorylate Aurora A at T288, the key regulatory site for vertebrate Aurora A activation (Scutt et al., 2009; Sloane et al., 2010). This phospho site was identified by Western blotting with a phosphospecific pT288 Aurora A antibody (Fig. S5 C) and also by mass spectrometry (Fig. S5, F–K). Critically, we found that dephosphorylated (low-activity) Aurora A was reactivated by incubation with catalytically active Plk4 (but not kinase-dead D154A Plk4) nearly as efficiently as it was by TPX2 (Fig. S5 D), an allosteric activator of Aurora A (Eyers et al., 2003). Consistently, we also observed that preincubation with Plk4 and ATP led to a time-dependent increase in Aurora A activity (Fig. S5 E). Thus, although Plk4 and Aurora A can have independent targets, these experiments suggest that the enhancement of Aurora A function in the oocyte could in part be explained through concerted activation of Aurora A by Plk4. This is unlikely to be the whole story, however, because TACC3 phosphorylation is reduced after inhibition of Aurora A, but not Plk4 (Fig. 4, A and F), This suggests that the two kinases might participate in distinct regulatory pathways. It also implies that Aurora A is autoactivated or activated by kinases or cofactors that are distinct from Plk4 in vivo (see Fig. 10). Collectively, our findings suggest that both Plk4 and Aurora A have independent and interdependent roles at MTOCs and in the vicinity of chromosomes to nucleate microtubules and initiate spindle formation.

### Aurora A, Plk4, and Ran-GTP differentially control microtubule growth

**Figure 6.**
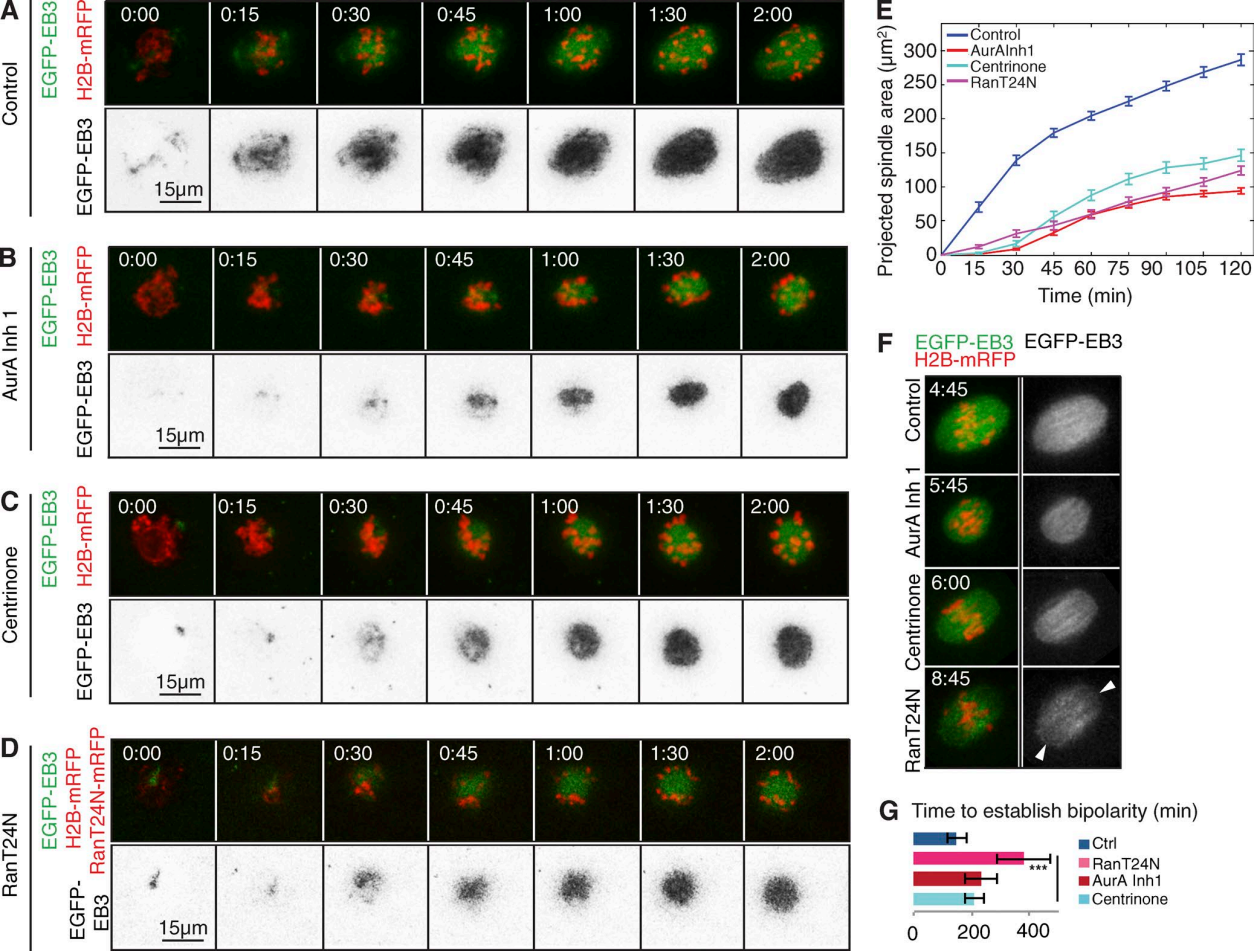
**Ran, Plk4, and Aurora A cooperate to promote bipolar spindle assembly.** (A–D) Time-lapse series of oocytes injected with mRNA encoding EGFP-EB3 (green, top; inverted monochrome, bottom) and H2B-mRFP (red, top). Progression through meiosis I is shown for controls (A), oocytes treated with 1 µM Aurora A inhibitor 1 (AurA Inh 1; B), oocytes imaged in the presence of 2 µM of the Plk4 inhibitor centrinone (C), and oocytes coinjected with RanT24N-mRFP (D). (E) Quantification of the size of the projected spindle area over time. RanT24N, Plk4, and Aurora A alone each contribute to meiotic spindle assembly, because their inhibition causes significantly reduced microtubule growth. Error bars indicate SEM. For p-values, see Table S1. (F) Stills of time-lapse movies showing metaphase spindles in oocytes undergoing meiosis I in conditions described in A–D. RanT24N, as opposed to Aurora A or Plk4 inhibition, affects spindle pole integrity (arrowheads). (G) Compared with Aurora A and Plk4 inhibition, dominant-negative Ran (RanT24N) significantly increases the time taken to establish bipolarity. Error bars indicate SEM.

Our findings suggested that Aurora A and Plk4 could together contribute to microtubule nucleation in the oocyte in addition to Ran. This led us to compare the effects of inhibiting these three pathways. As before, we used Aurora A inhibitor 1 and centrinone to inhibit Aurora A and Plk4 protein kinases, and we suppressed the Ran pathway using dominant-negative (T24N) Ran. We found that all three treatments led to a similar reduction of microtubule nucleation in the 2-h interval after NEBD (Fig. 6, A–E). However, notably distinct from kinase inhibition, Ran T24N led to loss of spindle pole integrity (Fig. 6 F) and also resulted in a significant delay in the establishment of spindle bipolarity (Fig. 6 G). This suggests that in addition to the initial diminution of microtubule density after NEBD, the Ran pathway might have other functions beyond those of Aurora A and Plk4 that could possibly reflect other downstream effectors of Ran-GTP.

**Figure 7.**
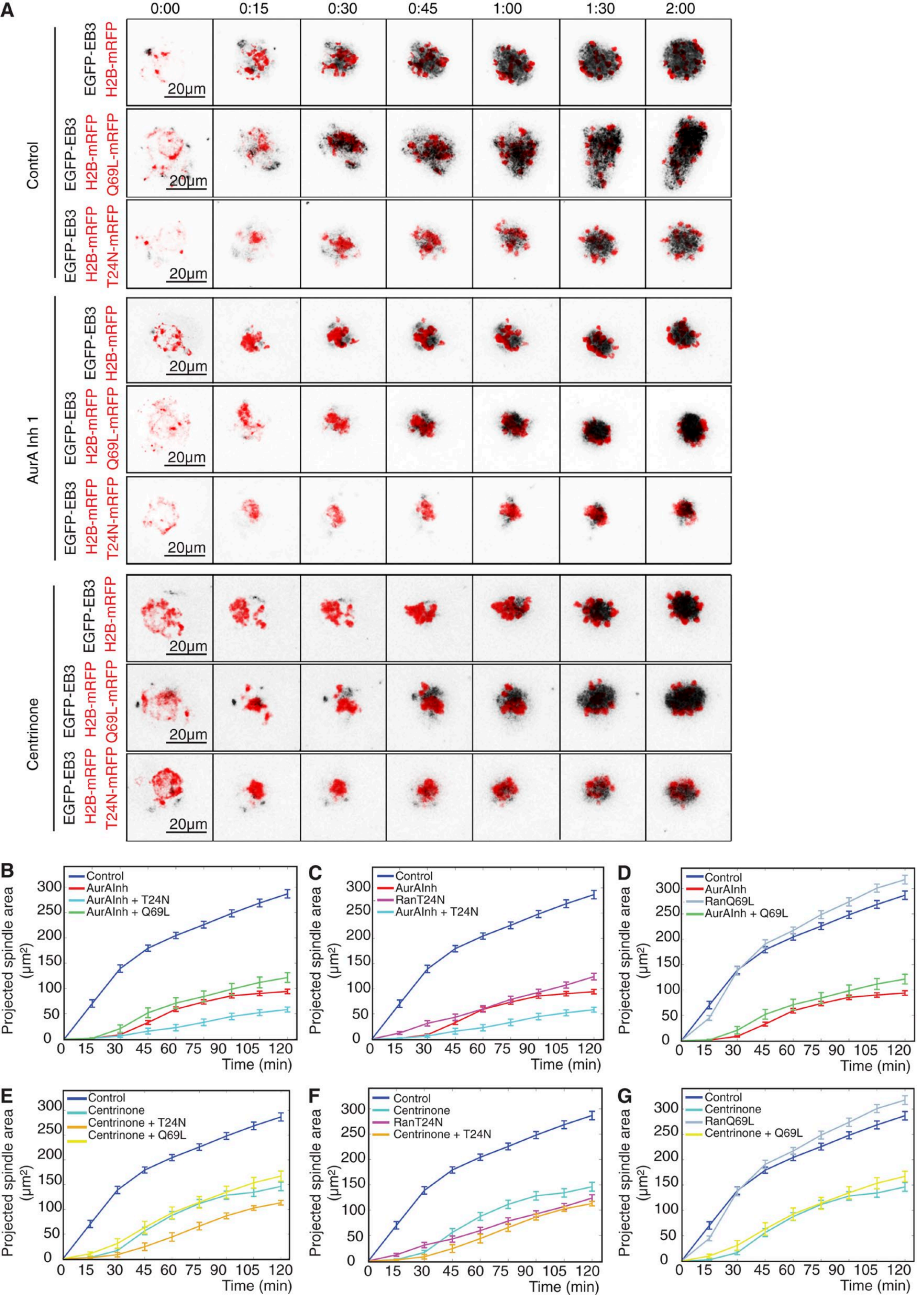
**Ran has distinct functions from Aurora A and Plk4 during assembly of a meiosis I spindle.** (A) Time-lapse imaging of oocytes injected with EGFP-EB3 (black, inverted) and histone H2B-mRFP (red) mRNAs. Shown are controls and oocytes treated with 1 µM Aurora A inhibitor 1 or 2 µM centrinone. For each condition, oocytes were additionally injected with mRNA encoding dominant-negative RanT24N-mRFP or constitutively active RanQ69L-mRFP. (B–G) Size of projected spindle areas. In untreated control conditions, RanQ69L expression (D and G; *n* = 22) significantly increases EGFP intensities, whereas RanT24N (C and F; *n* = 18) decreases these values compared with controls (*n* = 44). Compared with oocytes treated with Aurora A inhibitor 1 (AurA Inh 1) alone (B–D; *n* = 21), RanT24N in the presence of Aurora A inhibitor 1 (B and C; *n* = 13) causes a further reduction in the projected spindle area; RanQ69L (D; *n* = 13) is not able to significantly rescue these defects. Under inhibition of Plk4 by centrinone treatment (E–G; *n* = 23), RanT24N (E and F; *n* = 12) induces a further depression of quantified spindle area throughout time; RanQ69L (G; *n* = 13) leads to an increase of these values at later time points. Error bars indicate SEM. See also Video 4.

We then asked whether expression of the gain-of-function mutant Ran Q69L might overcome the effects of inhibiting either Aurora A or Plk4. By itself, Ran Q69L led to an increase in projected spindle area, in accord with Ran’s effect on microtubule density (Fig. 7, A, D, and G; and Video 4), but was unable to overcome the delay in microtubule nucleation in the 30 min after inhibition of either Aurora A or Plk4. However, once some microtubules had formed, Ran Q69L promoted an increase in microtubule growth to levels above those of untreated control oocytes (Fig. 7, A and D; and Video 4). The effects of inhibiting either kinase combined with the expression dominant-negative Ran T24N was additive and resulted in greatly diminished microtubule nucleation after NEBD (Fig. 7, A, B, D, E, and G; and Video 4). Together, these data suggest that Ran is able to play a role beyond that of Aurora A and Plk4 in initiating microtubule nucleation after the resumption of female meiosis.

**Figure 8.**
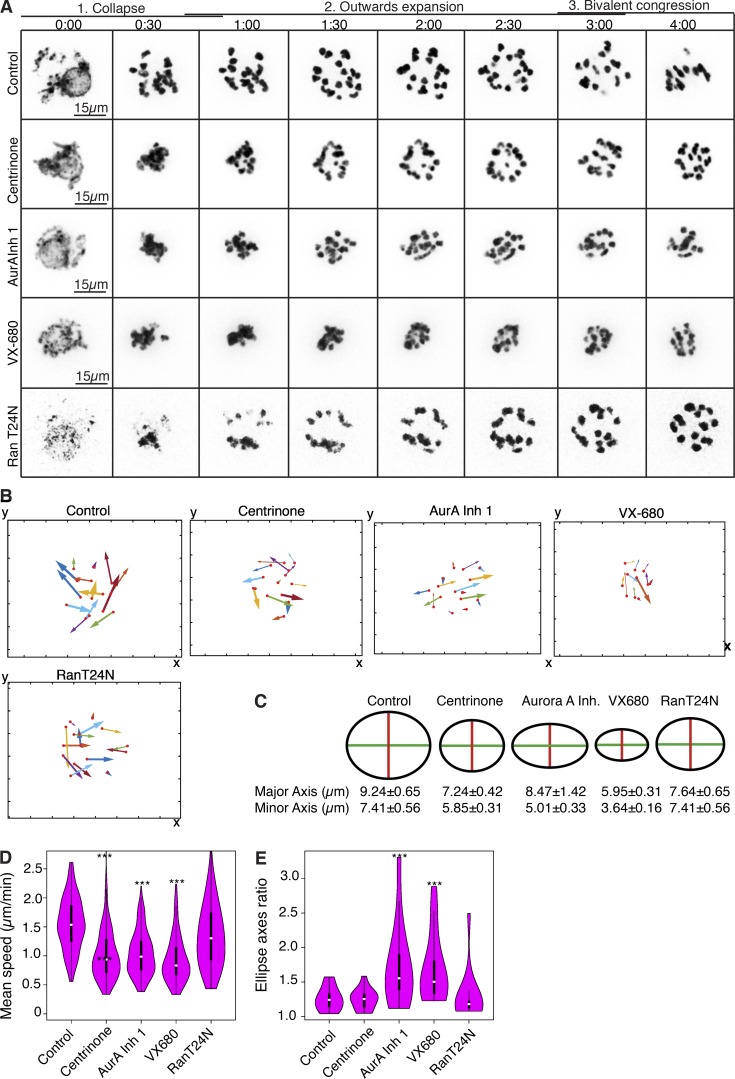
**Cooperation among Ran, Aurora A, and Plk4 in regulating bivalent movement and congression toward the metaphase I plate.** (A) Time-lapse imaging of oocytes expressing histone H2B-mRFP (inverted, black). Shown are the first 4 h of meiosis I after NEBD for controls; oocytes treated with 2 µM centrinone, 1 µM Aurora A inhibitor 1 (AurA Inh 1), or 1 µM VX-680; and oocytes expressing dominant-negative RanT24N. Three phases of chromosome movement (collapse, outward expansion, and bivalent congression) are indicated. (B) Representative chromosome tracks of the outward expansion for each indicated condition. Directionality and distance of movement is represented by the angle and length of the arrows, respectively; arrow thicknesses illustrate speeds. (C) Schematic representation of chromosome distributions at their maximum expansion, as quantified in E. Mean values for major and minor axes for each condition are shown (±SEM). (D) Violin plots depicting the frequency distribution of mean velocity of chromosome movements, which is significantly reduced as a result of Plk4 and Aurora A inhibition. (E) Fitting of the chromosome end coordinates at their maximal expansion to an ellipse. Control, RanT24N-expressing, and centrinone-treated samples display an isotropic distribution of chromosomes that tends to a 2D circle (ratio between major and minor axis approximates 1). In oocytes treated with Aurora A inhibitor 1 or VX-680, chromosomes show a preferential expansion direction along the axis of spindle, indicated by a ratio between major and minor axis of the fitted ellipse that is higher than 1 and significantly differs from the control (see Materials and methods; ***, P < 0.001).

As an alternative approach to monitoring the effects of inhibiting the Aurora A, Plk4, and Ran pathways, we followed the movement of chromosomes, which is mainly promoted by microtubule growth in the earliest steps of spindle formation. We could distinguish three broad dynamic phases of chromosome movement: (1) an inward movement within the first 30–45 min of NEBD as condensing chromosomes collapsed onto each other when there is very little microtubule growth, (2) an outward expansion of the chromosomes that occurs between 45 min and 2 h:30 min after NEBD, and (3) the onset of chromosome congression onto the metaphase plate (Fig. 8 A). Because the outward expansion process represents chromosomes being forced apart by the increase in number and length of growing microtubules, we focused on this specific phase.

Our movies (Fig. 8 A) indicated that the extent and velocity of movement was decreased upon inhibition of either Aurora A or Plk4 (Fig. 8, A, B, and D), and this was more pronounced when both kinases were inhibited simultaneously by VX-680. Chromosome expansion was least affected by the dominant-negative Ran T24N mutant. We also observed that Aurora A inhibition appeared to allow chromosomes to adopt a bipolar pattern of movement (Fig. 8, B and D). To assess further this directionality of movement, we represented the distribution of the 2D coordinates of chromosomes within an ellipse (Fig. 8, C and E). The relative mean dimensions of the axes of such ellipses indicate that inhibition of Plk4 or Aurora A reduced chromosome expansion to a similar extent that appeared additive on dual inhibition. The axial ratio of the ellipses indicated that chromosomes expand as a spherical ball in control oocytes and after Plk4 inhibition but more elliptically after Aurora A inhibition (Fig. 8, C and E). This difference in expansion pattern of the chromosome mass after inhibition of Aurora A accords with a role for Aurora A in the promotion of microtubule growth around chromatin and a need for Aurora A activity to establish the correct number and size of MTOCs (Fig. 4).

The pattern of chromosome movement after disruption of the Ran pathway indicated that the dominant-negative RanT24N did not reduce the velocity of the outward expansion of the chromosome mass at NEBD to the same extent as Aurora A inhibitor 1 and centrinone. Down-regulation of Ran function also had little effect on the spherical organization of the chromosome mass as it expanded in comparison to inhibition of Aurora A (Fig. 8, C and E). This points toward an independent function of the Ran pathway, as previously suggested.

**Figure 9.**
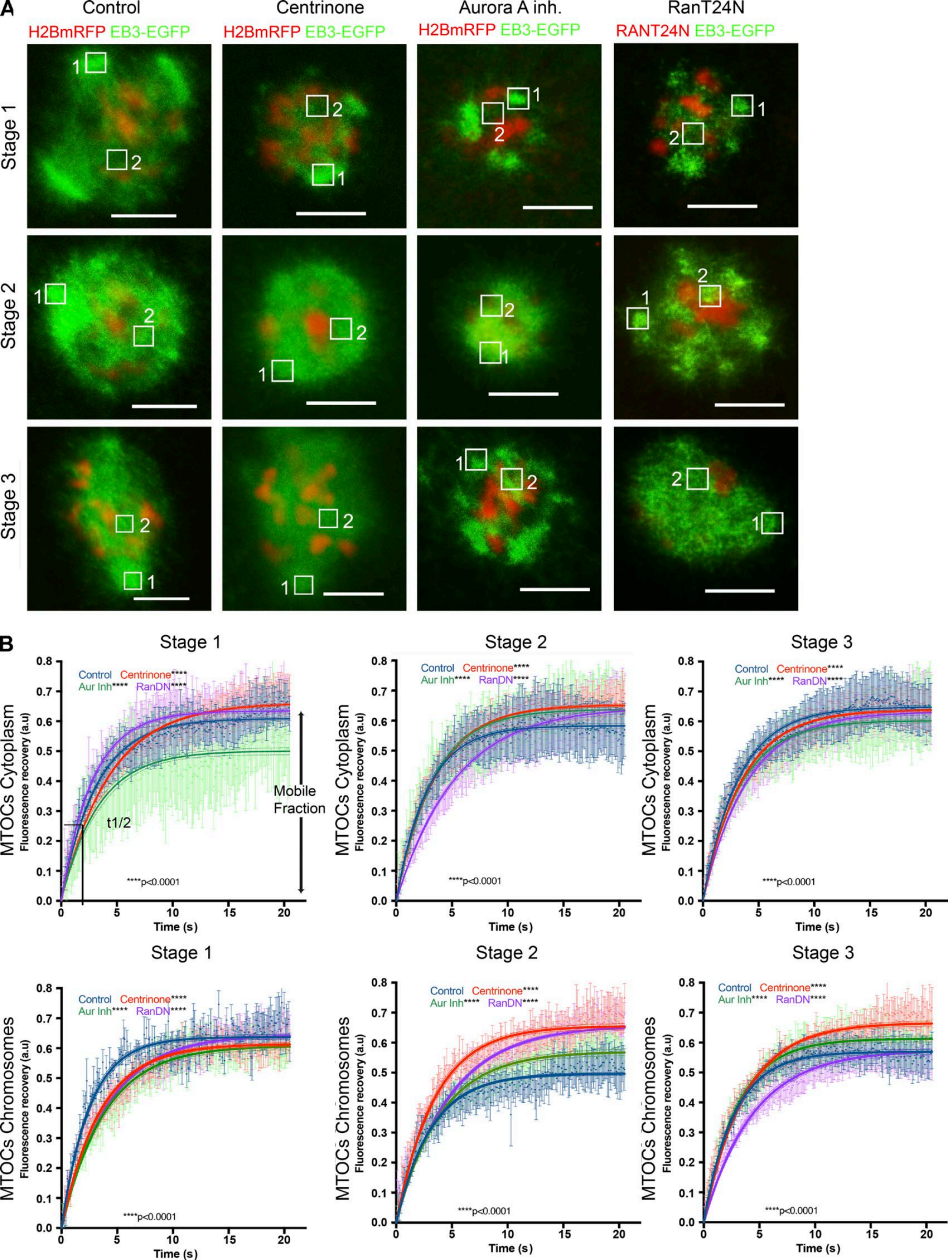
**Temporal and spatial analysis of Aurora, Plk4 and RAN in the early steps of microtubule growth and dynamics.** (A) Representative time-lapse images of oocytes injected with EGFP-EB3 (green) and histone H2B-mRFP (red) mRNAs showing the three different stages after oocyte meiosis I resumption under four experimental conditions: standard control conditions, Plk4 inhibition by addition of 2 µM centrinone, Aurora A inhibition by addition of 1 µM Aurora A inhibitor I, and Ran pathway inhibition by coexpression of the RanDN-negative form RanT24N. Regions of interest (ROIs) are indicated in the image, where ROI 1 is marking a cytoplasmic MTOC and ROI 2 a chromosomal MTOC typically bleached and analyzed by FRAP. Stage 1 occurs immediately after NEBD for a period of 30–45 min, during which time microtubules emanating from cytoplasmic MTOCs and around chromosomes are observed. Stage 2 comprises the time when microtubule density increases adopting a round ball-like shape. Stage 3 is the early phase of bipolarization when the spindle begins to elongate and MTOCs cluster toward the poles. Bars, 10 µm. (B) Graphs show the nonlinear fit curve for EB3-EGFP fluorescence intensity after bleaching (see Materials and methods for details) in oocytes after meiosis resumption under the different experimental conditions through the three stages described in A. Time after photobleaching is indicated as *t* = 0 s Fluorescence recovery and *t*_1/2_ were determined by applying a single exponential fitting to the recovery curve. Time is given in seconds. Each curve represents the mean value and 95% confidence interval. The 95% confidence interval for mobile fraction and *t*_1/2_ are shown below each graph. Plot of mean of values for each dataset at the three different stages are also shown. Error bar represents SEM. See also Table S4. These samples were both tested for normally distributed (D’Agostino–Pearson) and nonparametric tests (Wilcoxon matched pair test). In both cases, the null hypothesis was rejected (***, P < 0.001).

To gain insight into whether the different effects of inhibiting Aurora A, Plk4 or Ran might affect microtubule stability or nucleation, we assessed FRAP under these three conditions in oocytes expressing EB3-EGFP. We classified oocytes resuming meiosis I into three different stages (1–3; Fig. 9 A), as we did when assessing chromosome movement previously, and simultaneously bleached EB3 fluorescence both close to cytoplasmic MTOCs and in the vicinity of chromosomes (Fig. 9 A). We then followed fluorescence recovery in these two regions of interest (ROIs; 1 and 2).

The striking feature of stage 1 was that the recovery of fluorescence in the regions of cytoplasmic MTOCs fluorescence was only ∼50% if Aurora A was inhibited. This indicates a higher immobile population of EB3 compared with either control oocytes (60%) or those treated to inhibit Plk4 or Ran. A higher immobile population suggests that microtubules growing from cytoplasmic MTOCs are less dynamic after Aurora A inhibition at this very early stage (Figs. 9 B and Table S4). This effect of Aurora inhibition was not seen in stage 2 between 45 min and 2 h:30 min after NEBD, when the most pronounced effect was a slowing of the rate of fluorescence recovery after expression of dominant-negative Ran or Plk4. As the bipolar spindle began to form in stage 3, there were no significant differences in fluorescence recovery around cytoplasmic MTOCs in control oocytes or oocytes that had received any of the three treatments.

In assessing fluorescence recovery in the vicinity of chromatin, we saw little effect of the inhibitory treatments during stage 1. However, as microtubules adopted a ball-shaped arrangement in stage 2, all treatments led to an increased mobile fraction relative to untreated oocytes after Ran or Plk4 inhibition. This suggests destabilization and increased dynamicity of microtubule plus tips by these treatments. In addition, an effect upon the *t*_1/2_ for fluorescence recovery after all treatments suggests all three pathways are required for microtubule growth. An increase in *t*_1/2_ after Ran and Plk4 inhibition indicates the requirement of these two pathways to promote microtubule growth around chromatin. In stage 3, by the time bipolarity begins to be established, an increase in the mobile fraction around chromatin was seen after Aurora A or Plk4 inhibition and the loss of Ran function continued to depress the kinetics of fluorescence recovery, confirming its continued requirement to promote microtubule growth around chromatin and MTOCs.

Together, these findings point to distinct roles for Plk4, Aurora A, and Ran in directing the initial growth of microtubules upon meiotic resumption. The most pronounced effect of Aurora A inhibition was to reduce the dynamicity of microtubules around cytoplasmic MTOCs immediately after NEBD. Plk4 inhibition, on the other hand, led to loss of stability of microtubules in the vicinity of chromatin, particularly when microtubules were present as a ball-like mass. Inhibition of the Ran pathway depressed the growth of all microtubules but particularly in the vicinity of chromatin and as the bipolar spindle began to form. Such kinetics of fluorescence recovery in oocytes expressing dominant-negative Ran accord with a role for Ran in promoting microtubule growth after NEBD as found by Schuh and Ellenberg (2007) and point to Ran-GTP having cooperative but distinct roles from those of Aurora A and PLk4 in the initiation of spindle assembly in the oocyte.

## Discussion

Although the gradient of Ran-GTP around chromatin is critical to promote spindle formation in the absence of centrosomes in many systems (O’Connell and Khodjakov, 2007), it is not essential for formation of the first bipolar meiotic spindle upon meiotic resumption in the acentriolar mouse oocyte, even though it does contribute to the increase in density of microtubules after NEBD (Dumont et al., 2007; Schuh and Ellenberg, 2007). By analyzing the very first events in spindle formation upon NEBD, we find that two protein kinases, Aurora A and Plk4, both contribute to trigger microtubule growth and formation of the first meiotic spindle. Inhibition of either kinase dramatically reduces microtubule growth, leading to a reduction in the velocity of the outward expanding chromosomes that are pushed apart by nascent microtubules as the spindle forms. Plk4 inhibition diminishes microtubule growth upon resumption of meiosis, but bipolar spindle formation does eventually occur in a process that requires Aurora A, but not Plk1. The specific defects associated with the inactivation of Plk4 or Aurora A suggest that they have both independent and overlapping roles.

Independent functions of the two kinases are reflected in the different effects of their inhibition upon the organization of MTOCs, upon the expansion pattern of chromosomes after NEBD, and upon the recovery of fluorescence of a microtubule plus tip–associated protein after photobleaching. Together, these findings suggest that the two kinases affect microtubule growth in different ways. In the 30–45 min after NEBD, inhibition of either kinase severely reduces microtubule growth, but Aurora A inhibition also results in cytoplasmic MTOCs that are reduced in both size and number. The reduced recovery of EB3-GFP fluorescence after photobleaching in the vicinity of the cytoplasmic MTCs after Aurora A inhibition also suggests a loss of dynamicity of the nucleated microtubules not seen after Plk4 inhibition. Similarly the expansion of chromosomes associated with a growing ball of microtubules between 45 min and 2 h and 30 min after NEBD is greatly reduced by inhibition of either kinase. However, again the effect of Aurora A inhibition appears greater and results in a more pronounced bidirectional movement of chromosomes. This may reflect greater ease in establishing bipolarity from a reduced number of cytoplasmic MTOCs after Aurora A inhibition. In contrast, Plk4 inhibition leads to greater dynamicity of microtubules in the vicinity of chromosomes than does Aurora A inhibition.

The overlapping functions of the two kinases are indicated by the extent to which their drug-resistant variants can rescue the effects of their simultaneous inhibition by VX-680, a drug that almost completely suppresses microtubule growth after NEBD. This phenotype is substantially rescued by a drug-resistant form of Aurora A, but not by drug-resistant Plk4 alone. However, drug-resistant Plk4 can enhance the rescuing ability of drug-resistant Aurora A upon combined inhibition of the two endogenous kinases. This suggests that in addition to having independent functions, a subset of Plk4 functions might be dependent on Aurora A. This hypothesis is supported by the finding that Plk4 is able to phosphorylate Aurora A at T288 in its T-loop and is so able to potentiate its function (Fig. S5). The sequence surrounding T288 in the Aurora A activation segment conforms to a relaxed Plk4 substrate consensus and differs markedly from the acidic context required for Plk1–3 phosphorylation (Johnson et al., 2007). This is suggestive of a potential role for Plk4 in Aurora A regulation.

We also find a decrease in microtubule growth after NEBD after inhibition of the Ran pathway that accords with the findings of Schuh and Ellenberg (2007). The diminution of microtubule growth after inhibition of either Aurora A or Plk4 is accentuated by dominant-negative Ran but not rescued by constitutively active Ran. This suggests that the kinases and the Ran pathway function interdependently. The recovery of FRAP microtubule plus tip–associated EB3-GFP suggests that Ran is required for the kinetics of microtubule growth, particularly around cytoplasmic MTOCs as the microtubule ball expands and in the vicinity of chromatin as the bipolar spindle forms. Thus, although Ran is not required for bipolar spindle formation (Dumont et al., 2007), our findings provide support for its role in promoting microtubule growth (Schuh and Ellenberg, 2007) after NEBD and would accord with a role in releasing microtubule-associated proteins such TPX2 to promote microtubule stabilization at later stages (Brunet et al., 2008).

In contrast to the similar phenotypes that result from Aurora A or Plk4 inhibition in the oocyte, the consequences of inhibition of these kinases in the acentriolar mouse embryo are quite different. Notably, depletion of Plk4, expression of dominant-negative forms, or centrinone treatment in the acentriolar embryo (Fig. S1) result in a severe delay of microtubule nucleation, monopolar spindle formation, and cytokinesis defects (Coelho et al., 2013), whereas inhibition of Aurora A has only a slight effect on spindle size (Fig. S1). These differences between the oocyte and the embryo might reflect the profoundly different aspects of cell cycle regulation in these two acentriolar cell types. The prolonged nature of meiosis I with gradual activation of Cdk1 (Brunet and Maro, 2005) contrasts with the extremely rapid activation of Cdk1 during mitosis and meiosis II. As the dynamic instability of microtubules responds to Cdk1 activity, a dual requirement for cooperative Aurora A and Plk4 activities to initiate spindle formation could represent a conserved “fail-safe” mechanism to ensure rapid microtubule growth in the window of low Cdk1/cyclin B activity upon meiotic resumption.

**Figure 10.**
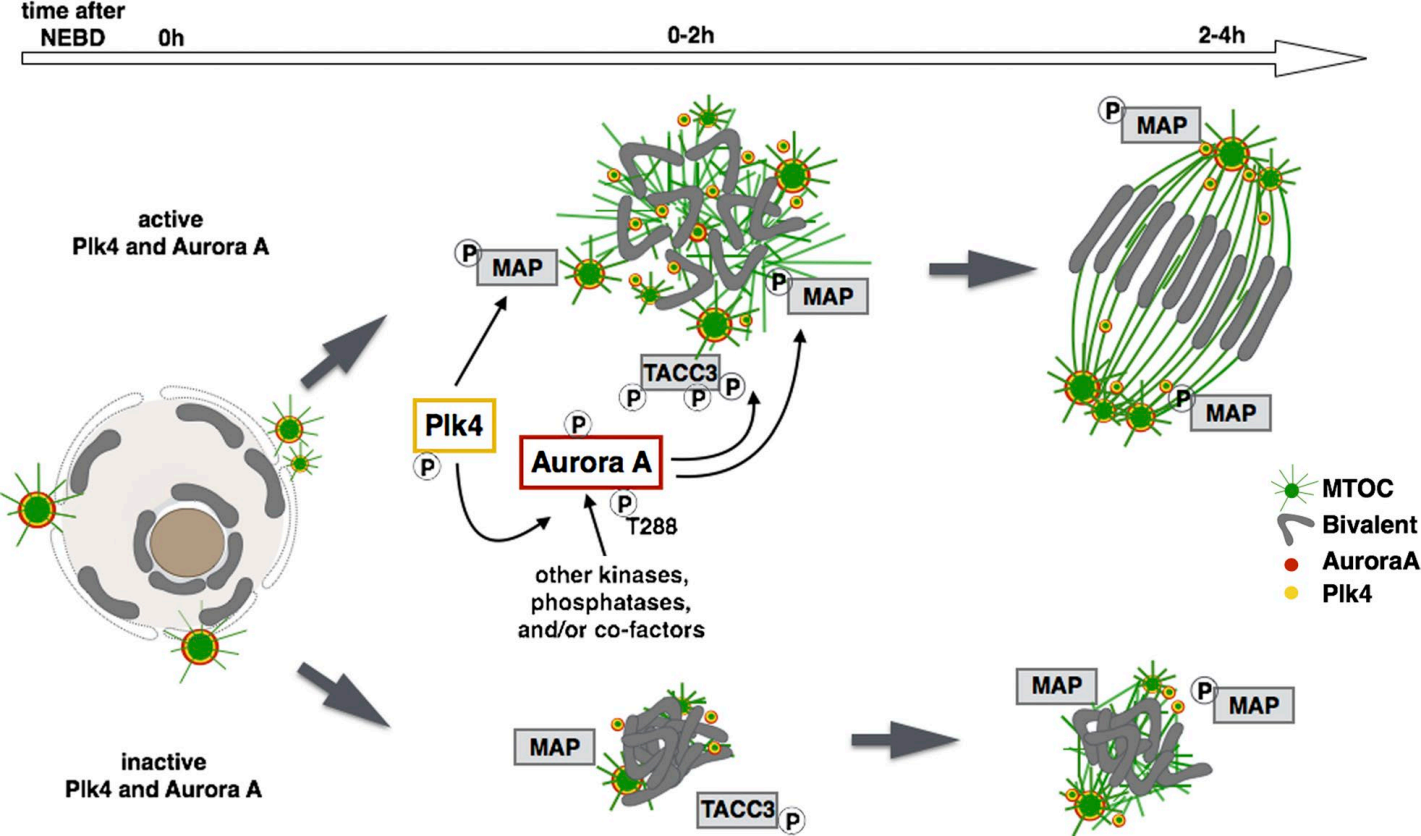
**Meiosis I spindle formation is a result of Aurora A, Plk4, and Ran-GTP activity.** Spindle assembly in the oocyte is a multistep process triggered by the cooperative activity of Plk4 (yellow) and Aurora A (red), which are essential to initiate microtubule growth at NEBD. Plk4 and Aurora A localize to MTOCs that are dispersed around condensed chromosomes. Aurora A activity is potentiated by Plk4, directly phosphorylating the kinase at five Ser/Thr residues including the T-loop activating site, but likely undergoing additional regulation by meiotic kinases and/or regulatory binding partners. Both Aurora A and Plk4 can have a distinct and shared set of substrates. We identify TACC3 as one potential downstream effector of Aurora A that localizes to MTOCs and whose phosphorylation is reduced upon Aurora A inhibition (see Fig. 4 C). Other pathways are likely acting at later stages of spindle assembly and are able to partially overcome the defects caused by inhibition of Aurora A and Plk4. One redundant pathway is Ran-GTP, which, by increasing levels of the allosteric Aurora A activator TPX2, is important for TACC3-mediated microtubule stabilization at the spindle poles.

In conclusion, we have identified Aurora A and Plk4 as protein kinases that act in concert with Ran to trigger rapid growth of microtubules to allow the assembly of a robust acentrosomal meiosis I spindle. Interestingly, Aurora A appears able to act as a potential Plk4 effector. However, it would seem that Plk4 has additional, currently unknown substrates that account for its functions that are independent of Aurora A. We anticipate that Aurora A can also be activated by other mechanisms in oocytes resuming meiosis. Indeed, we have identified TACC3, known to contribute to the activation of Aurora A, as one of Aurora A’s early targets at MTOCs (Fig. 10). The existence of multiple mechanisms for initiating spindle formation in the oocyte in the absence of centrosomes points to the critical need to safeguard the equitable transmission of chromosomes to the next generation, arguably the single-most important cell division in the vertebrate life cycle.

## Materials and methods

### Oocyte and embryo collection and culture

Oocytes were collected from 4- to 8-wk-old F1 females and recovered from tertiary follicles as previously described (Wianny and Zernicka-Goetz, 2000). Isolated oocytes were cultured under mineral oil in M2 media supplemented with 1 µM dbc-AMP (Sigma) to prevent meiotic maturation. During time-lapse imaging, oocytes were cultured in M16 medium (Sigma) without dbc-AMP at 37°C in a 5% CO_2_ atmosphere. For embryo microinjections, C57BL6 × CBA mice were mated after superovulation of females, and embryos were collected in M2 media following previously described standard procedures (Piotrowska-Nitsche et al., 2005; Bischoff et al., 2008). Isolated embryos were cultured in KSOM media under paraffin oil at 37°C in a 5% CO_2_ atmosphere. For the purpose of inhibitor studies, the media was supplemented with inhibitors at the following concentrations: 500 nM for MLN8237 (SelleckChem), 100 nM for BI2536 (SelleckChem), 1–5 µM for Aurora A inhibitor 1 (SelleckChem), 2–5 µM for centrinone (provided by Y. Liang Wong and K. Oegema, Ludwig Institute for Cancer Research, University of California, San Diego, San Diego, CA, and by T.C. Gahman and A.K. Shiau, Ludwig Institute for Cancer Research, San Diego, CA; described in Wong et al., 2015), 0.5–5 µM for VX-680, and 1 µM for saracatinib (Table S3). All experimentation with mice was performed following requirements of the UK Home Office under a project license held by M. Zernicka-Goetz.

### Microinjections

For microinjection of mRNA, in vitro transcription of Sfil-linearized RN3P plasmids was performed using mMessage mMachine T3 Polymerase (Life Technologies) according to the manufacturer’s instructions. Nhe1-linearized pGEM plasmid was in vitro transcribed using mMessage mMachine T7 Polymerase (Life Technologies). Microinjection of in vitro transcribed mRNAs was performed as previously established (Zernicka-Goetz et al., 1997). After injection, oocytes were left for 3–4 h before imaging. For microinjections of embryos, a single random blastomere was microinjected with the RNAs to monitor cell division at the two-cell stage.

### Live cell imaging and image analysis

Oocytes released from prophase arrest were selected manually, and only those oocytes included that harbored a surrounded nucleolus configuration (Zuccotti et al., 2005). NEBD was established as the time 00:00, as the time when EB3-GFP signal was first observed inside the nucleus, corresponding to the NEBD. Prometaphase I comprises this period until metaphase I, when bivalents assume an aligned position at the equatorial plate. At metaphase I, the distance between spindle poles is also reduced until the onset of anaphase I. This criterion was also used to help defining this stage. Cytokinesis I is defined when extrusion of the polar body is detected. Time-lapse images were collected every 15 min over the course of 20 h for the green and red channels on an inverted Zeiss Axiovert with a spinning disk confocal head (Intelligent Imaging Solutions) using a 63×/1.3 water objective. Each Z stack comprises 20 images at 3-µm intervals. Acquisition was performed using Slidebook software 6.0.0.20. Images were converted to 16-bit TIFF file format using ImageJ, and all image analysis was performed with the custom algorithms available in ImageJ analysis software (Schneider et al., 2012).

Quantitation of total α-tubulin by immunofluorescence indicated measurement of the space occupied by EGFP-EB3 to provide a suitable way of assessing spindle growth after centrinone inhibition of Plk4 (Fig. S4).

Detection of chromosomes, measurement of x–y coordinates for each time frame, and the mean speed of chromosome movement was performed using TrackMate plugin. Time frames that corresponded to the outward expansion were manually selected. The outward-expansion process was followed for 2 h, and only tracks that detected chromosomes for five to nine frames were included in the analysis. For EB3 intensity measurements, ROIs corresponding to the spindle area were manually selected. Pixel intensity in the spindle area and in an equivalent area in the cytoplasm (background fluorescence) were determined. The size of the spindle area was determined after image thresholding.

### Data preprocessing

Spindle-area data and EB3 intensity quantifications were initially corrected by subtracting background values. Time-course data were normalized to the first time point by subtracting the first value (time zero) from all other time point measurements. Resulting data for EB3 intensity and spindle area were plotted as mean ± SEM (error bars) for each time point.

### Statistical comparison between different conditions in each time point

For the pairwise comparison in each time point, we considered the set of preprocessed measurements of the two conditions to compare (i.e., control vs. condition, or two different conditions), and we assessed whether the difference was significant using the two-sided Wilcoxon rank-sum test.

### Analysis of spindle expansion kinetics

To describe the kinetics of microtubule and spindle growth in the different conditions, we assumed that spindle expansion follows a sigmoidal-shaped curve. The preprocessed EB3 intensity and spindle area quantifications were fitted to sigmoid curves using the following four-parameter logistic function:

$$y=A_{1}+\frac{A_{2}-A_{1}}{1+10^{\left(t50-x\right)*.slope}},$$ (1)

where t50 represents the time when the spindle reaches the 50% of its maximal expansion and slope represents the velocity of such a process (t50^2h^).

The parameter vectors used to initialize the fitting procedure were [*A*_1_, *A*_2_, *t*50, *slope*] = [min(*data_condition_*), max(*data_condition_*), 40, 0]. The estimation of parameters that best fit the nonlinear regression relative to each experimental condition was performed using the MATLAB iterative least-squares estimation routine.

To evaluate differences in the global kinetics of the process, we compared t50^2h^ and the relative intervals of confidence at 95% estimated during the fitting of Eq. 1. Expansions with t50^2h^ and relative intervals of confidence completely disjoint were considered statistically different. For p-values and t50^2h^ values, see Tables S1 and S2, respectively.

### Analysis of the maximal spindle expansion pattern

We assumed spindle expansion is an isotropic process. The different conditions examined reduced spindle progression and expansion, resulting in a final anisotropic distribution of chromosomes at the maximal spindle expansion. To evaluate quantitatively such aspect in each individual experiment, we considered all quantifications relative to the final and maximal spindle expansion in terms of chromosome 2D coordinates, and we used them to fit an ellipse curve. In the control case the isotropic distribution of chromosome at the maximal expansion tends to a 2D circle (i.e., major and minor axes of the elliptic curve are comparable and their ratio tends toward 1). In case of a preferential direction expansion, the final spindle shape appears more elliptical, and therefore the ratio between major versus minor axis deviates from 1. Using the MATLAB-implemented function fit ellipse, the major and minor axes of the elliptic cloud of chromosomes were estimated given the x and y coordinates of the chromosomes at the maximal spindle expansion. The ratio between the major and the minor axes was then computed for each experiment, and then experiments of each condition were pooled to build a distribution. We could then assess differences in expansion pattern by comparing distributions relative to different conditions. To statistically assess differences between distributions, we used a MATLAB implementation of the Wilcoxon rank-sum test.

### cDNA constructs

We used the previously described constructs for EGFP-mPlk4-pRN3P; hPlk4-mcherry-pRN3P, EGFP-ΔKinasePlk4-pRN3P, EGFP-Plk4T170A-pRN3P, EGFP-EB3pRN3P, H2B-mRFP-pRN3P (Coelho et al., 2013), and Aurora B-pRN3P (Sharif et al., 2010). Mouse Aurora A (clone IOM16362) was subcloned in frame with EGFP at the 3′ end into RN3P vector for in vitro transcription of mRNA. The plasmid for p150CC1 was a gift from X. Zhu (Shanghai Institute for Biological Sciences, Chinese Academy of Sciences, Shanghai, China). p150CC1 was subcloned into pRN3P for in vitro transcription.

### Site-directed mutagenesis

For mutagenesis, the QuikChange II XL Site-Directed Mutagenesis kit (Stratagene) was used according to the manufacturer’s instructions. Oligonucleotides used are described in Table S5.

### In vitro kinase assays

1.5 µg purified “kinase-dead” N-terminally 6His-tagged D274N Aurora A was incubated with 3 µg of N-terminally 6His-tagged WT Plk4 (1–285) lacking the polo box domains or a catalytically inactive D154A Plk4 (1–264) mutant (±10 µM centrinone) at 37°C in 50 mM Tris, pH 7.4, 10 mM MgCl_2_, 1 mM DTT, and 1 mM ATP. Aliquots were removed at 0, 10, 30, 60, 120, and 240 min after reaction initiation and stopped by boiling in SDS sample buffer. To evaluate site-specific Aurora A phosphorylation, trypsin proteolysis and liquid chromatography–tandem mass spectrometry (LC-MS/MS) analysis was performed at the 120-min time point.

### Western blotting

In vitro kinase assays were analyzed by SDS-PAGE, after transfer onto a nitrocellulose membrane and probing with anti–Aurora A pThr288 antibody (1:5,000) overnight at 4°C. After washing with TBST and incubation with goat anti–rabbit IgG (1:5,000) for 1 h, pT288 Aurora A was visualized using ECL reagent. Equal loading of Aurora A D274N, WT and D154A Plk4 (1–285) was confirmed by Ponceau S staining of the membrane.

### EZ Reader kinetic kinase assays for Aurora A

The Caliper LabChip EZ Reader platform measures enzyme activity by assessing the mobility shift of a fluorescently labeled peptide substrate, which changes upon phosphorylation and can be quantified by comparative integration of phosphorylated and dephosphorylated peptide peaks. To assess the effects of GST, GST-TPX2, WT PLK4, or kinase-dead (D154A) PLK4 on inactive Aurora A, a 10-fold molar excess of each protein was incubated with 100 ng lambda-phosphatase–treated Aurora A with 1 mM ATP (to mimic the cellular concentration) and 10 mM MgCl_2_ for 30 min at 30°C. Aurora A activity was then assessed in kinetic mode by the addition of a fluorescent kemptide-derived peptide substrate (5FAM-Leu-Arg-Arg-Ser-Leu-Gly, with calculation of peptide phosphorylation every minute for 30 cycles at 20°C (1 min, approximately one cycle). To measure enhanced effects of WT PLK4 (1–285) on the activity of catalytically active (non–phosphatase treated) Aurora A, 2 µM of the Aurora A fluorescent kemptide-derived peptide substrate was incubated with 30 ng PLK4 and 10 ng Aurora A in 25 mM Hepes, 10 mM MgCl_2_, and 0.001% (vol/vol) Brij 35 buffer, with 1 mM ATP. Reactions were preincubated for 15 min at 37°C to permit activation of Aurora A, and measurements were taken over 60 EZ Reader cycles at room temperature in kinetic mode.

### Sample preparation for LC-MS/MS analysis

Disulfide bonds were reduced by addition of 3 mM DTT in 50 mM ammonium bicarbonate and heated at 60°C for 10 min. The resulting free cysteine residues were alkylated with 14 mM iodoacetamide (in the dark, at room temperature, for 30 min) and excess iodoacetamide quenched by addition of DTT to a final concentration of 7 mM. Proteins were digested overnight with trypsin (2% wt/wt) at 37°C.

### Mass spectrometry analysis

Nano-liquid chromatography electrospray ionization tandem mass spectrometry analysis was performed using a Thermo Fusion mass spectrometer attached to a Waters nanoAcquity ultra-high performance liquid chromatography system. Peptides were loaded onto the trapping column (PepMap100, C18, 300 µm × 5 mm; Thermo Scientific), using partial loop injection, for 7 min at a flow rate of 9 µl/min with 2% MeCN and 0.1% (vol/vol) trifluoroacetate and then resolved on an analytical column (Easy-Spray C18 75 µm × 500 mm 2-µm-bead-diameter column) using a gradient of 96.2% A (0.1% formic acid) and 3.8% B (80% MeCN, 19.9% H_2_O, and 0.1% formic acid) to 50% B over 30 min at a flow rate of 300 nl/min^−1^. A full-scan mass spectrum was acquired over *m/z* 400–1500 in the Orbitrap (60 K resolution at *m/z* 200) and data-dependent MS/MS analysis performed using a top-speed approach (cycle time of 3 s), using either higher-energy collisional dissociation (HCD) and/or electron-transfer and higher-energy collision dissociation (EThcD) for fragmentation, with product ions being detected in the ion trap (rapid mode). .raw files were converted to .mgf files in Proteome Discoverer. HCD and EThcD spectra were separated according to electron-transfer dissociation reaction time (<39 ms selects HCD spectra) generating two separate .mgf files. Using an in-house built Perl script, the two .mgf files were merged and searched using MASCOT against the *Escherichia coli* IPI database, with the sequences of the human Aurora A and Plk4 constructs as targets. Parameters were set as follows: MS1 tolerance of 10 ppm, MS/MS mass tolerance of 0.6 D, carbamidomethylation of Cys as a fixed modification, and phosphorylation of Ser and Thr, and oxidation of Met as variable modifications. The tandem MS data for the identified phosphopeptides was interrogated manually.

### Immunofluorescence and antibodies

Oocytes were fixed in ice-cold methanol/DMSO (9:1) for 30 min, followed by permeabilization in 1× PBS 0.1% BSA, 10% FBS, and 0.5% Triton X-100 for 1 h and blocking in 1× PBS, 0.1% BSA, 0.1% Tween, and 10% FBS for another 1 h. Incubation in primary and secondary antibodies was performed in 1× PBS, 0.1% BSA, 0.1% Tween, and 10% FBS. Washes were performed using 1× PBS, 0.1% BSA, and 0.1% Tween. Oocytes were mounted in Vectashield Mounting Medium with DAPI (Vector Laboratories). Images were collected on an SP5 or SP8 (Leica) with 63×/1.4 oil objectives using the Application Suite X software (LAS-X; Leica). Images were deconvolved using Huygens Professional software; processing and analysis was performed with ImageJ Version 1.45 s and Photoshop CS5 (Adobe). All images shown are the projections of optical sections.

We used the following antibodies: rat anti–mouse PLK4 (described in Coelho et al., 2013; 1:1,000); rat anti–α-tubulin-YL1/2 (1:50; Oxford Bioscences); mouse anti–α-tubulin-DM1A (1:10,000; Sigma); mouse anti–γ-tubulin-GTU88 (1:50; Sigma); rabbit anti–mouse Nedd1 (gift from J.A. Manning, Centre for Cancer Biology, Adelaide, Australia; described in Manning et al., 2008; 1:500); rabbit anti–human Cep192 (gift from L. Pelletier, Mt. Sinai Hospital, Toronto, ON, Canada; described in Zhu et al., 2008; 1:500); rabbit anti-phospho-Ser626 xTACC3 (gift from A.A. Hyman, Max Planck Institute of Molecular Cell Biology and Genetics, Dresden, Germany; described in Kinoshita et al., 2005; 1:750), and rabbit anti–human pAurAT288 (described in Tyler et al., 2007; 1:1,000).

The secondary antibodies used (1:1,000) were conjugated with Alexa Fluor 488, 568, or 647 (Invitrogen) and had minimal cross-reactivity to other species.

### FRAP conditions and analysis

FRAP was performed with a spectral confocal (SP8; Leica) with a 63×/1.4 NA objective lens and an additional zoom of 4.5×. Images were acquired every 10 ms or 1 s. Bleaching was conducted for 10 frames after five frames of prebleach imaging. 200 postbleaching images were collected for 20.698 s. Bleaching was performed with laser lines 476, 488, and 496 nm at 100% intensity and FRAP booster to expand the beam.

Pre- and postbleaching images were acquired with laser 488 nm at 4.9% and laser 561 nm at 8.6%, under bidirectional XYZT scanning mode at 1,400 Hz.

Two types of ROIs were obtained for normalization: the reference ROI to measure a decay in fluorescence caused by the acquisition bleaching and the base ROI to establish the background of the oocyte. ROIs were always of the same area and shape as the ROIs used for photobleaching (9.1 µm^2^). Normalization for bleaching was performed for each individual experiment. All FRAP experiments were performed at least three times. Replicates were biological replicates from different oocytes.

The values for fluorescence recovery at each time point were fitted to this nonlinear curve: *y* = *Ymax*[1 − *e*^(−*x* * *tau*)], where *t*_1/2_ represents the time when the fluorescence recovery reaches the 50% of its maximal expansion, and tau represents the velocity of such a process:

$$t_{1/2}=\frac{-\ln\left(0.5\right)}{tau}.$$

The parameter vectors used to initialize the fitting procedure were [*Ymax*,*Tau*] = [1,10], where x corresponds to time and Y each individual normalized value of fluorescence.

Sample size for each experiment is given in Table S4. Data are shown as mean and 95% confidence intervals. Statistical significance was evaluated using Prism 7 (GraphPad software).

### Online supplemental material

Fig. S1 shows that Plk4 inhibition by centrinone in the two-cell embryo prevents bipolar spindle formation. Figs. S2 and S3 show that Plk4 and Aurora A facilitate microtubule nucleation upon meiotic resumption. Fig. S4 shows that VX-680–mediated inhibition of Plk4 and Aurora A diminishes microtubule nucleation. Fig. S5 shows that Plk4 phosphorylates Aurora A on five phosphosites in vitro. Table S1 lists p-values for the size of the projected spindle area. Table S2 shows values of t50 and of the slope together with their relative confidence intervals. Table S3 shows inhibitor concentrations. Table S4 indicates FRAP values for *t*_1/2_, mobile fraction, and their relative confidence intervals. Video 1 shows that Plk4 is required for acentriolar spindle assembly and relates to Fig. 1. Video 2 shows that Aurora A plays a role in spindle assembly and relates to Figs. 2 and S2. Video 3 shows that Aurora A predominates over Plk4 to initiate microtubule nucleation and relates to Fig. 5. Video 4 shows that Ran has distinct functions from Aurora A and Plk4 during spindle assembly of meiosis.

## Supplementary Material

Supplemental Materials (PDF)

Video 1

Video 2

Video 4

Video 3
