# Supplementary material for: Plk4 and Aurora A cooperate in the initiation of acentriolar spindle assembly in mammalian oocytes
Source: J Cell Biol. 2017 Nov 6;216(11):3571–90. doi: 10.1083/jcb.201606077 (PMC5674873; doi:10.1083/jcb.201606077)
Supplement: Supplemental Materials (PDF) [file JCB_201606077_sm.pdf]

Bury et al., https://doi.org/10.1083/jcb.201606077

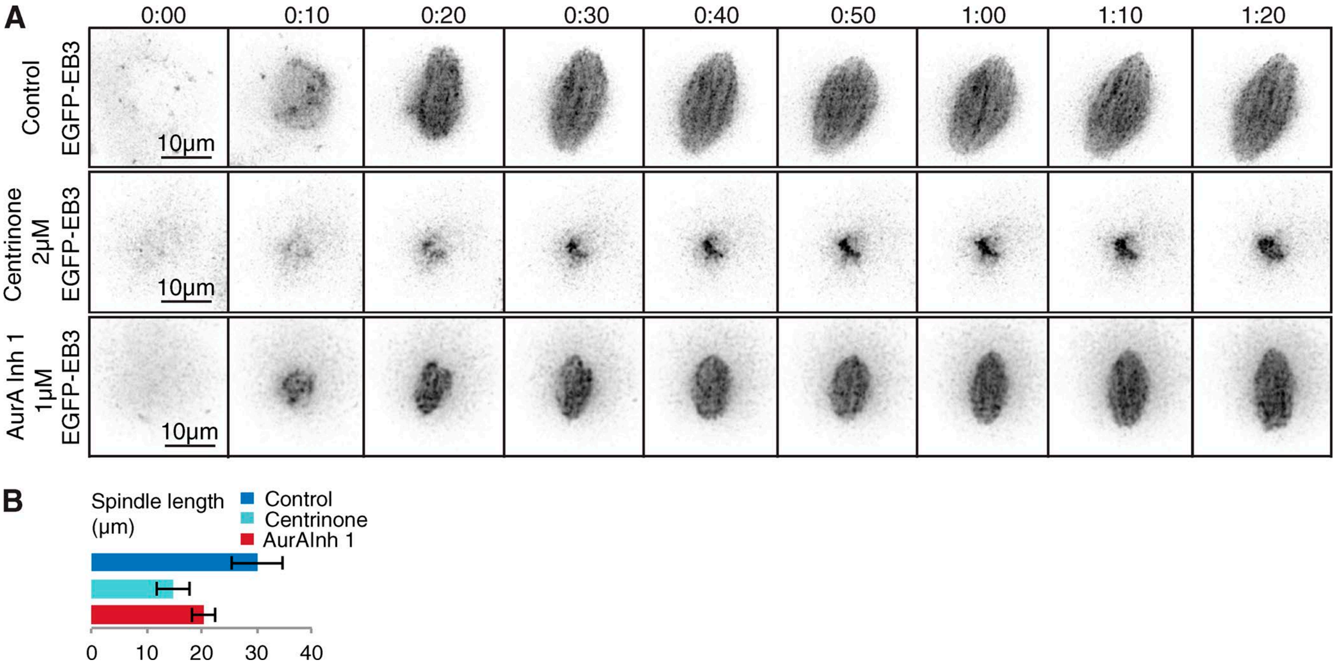

Figure S1. **Plk4 inhibition by centrinone in the two-cell embryo prevents bipolar spindle formation.** (A) Two-cell embryos injected with mRNA codifying EGFP-EB3 (black, inverted). Embryos were imaged for 10–16 h until spindle assembly occurred in standard untreated conditions (control, top), in the presence of 2 µM centrinone (middle), and in the presence of 1 µM Aurora A inhibitor 1 (bottom). Inhibition of Plk4 by the addition of centrinone affects spindle assembly, which is severely impaired compared with both control and Aurora A inhibition. However, the latter treatment leads to formation of a smaller spindle compared with controls. (B) Mitotic spindle length at metaphase in two-cell embryos. The quantification was performed for controls (*n* = 16), after Plk4 kinase inhibition (centrinone, *n* = 19), and after Aurora A inhibition (AurA Inh 1, *n* = 12). Error bars indicate SD of the mean. Differences between each individual inhibitor and control conditions are statically significant (***, $P < 0.0001$).

THE JOURNAL OF CELL BIOLOGY

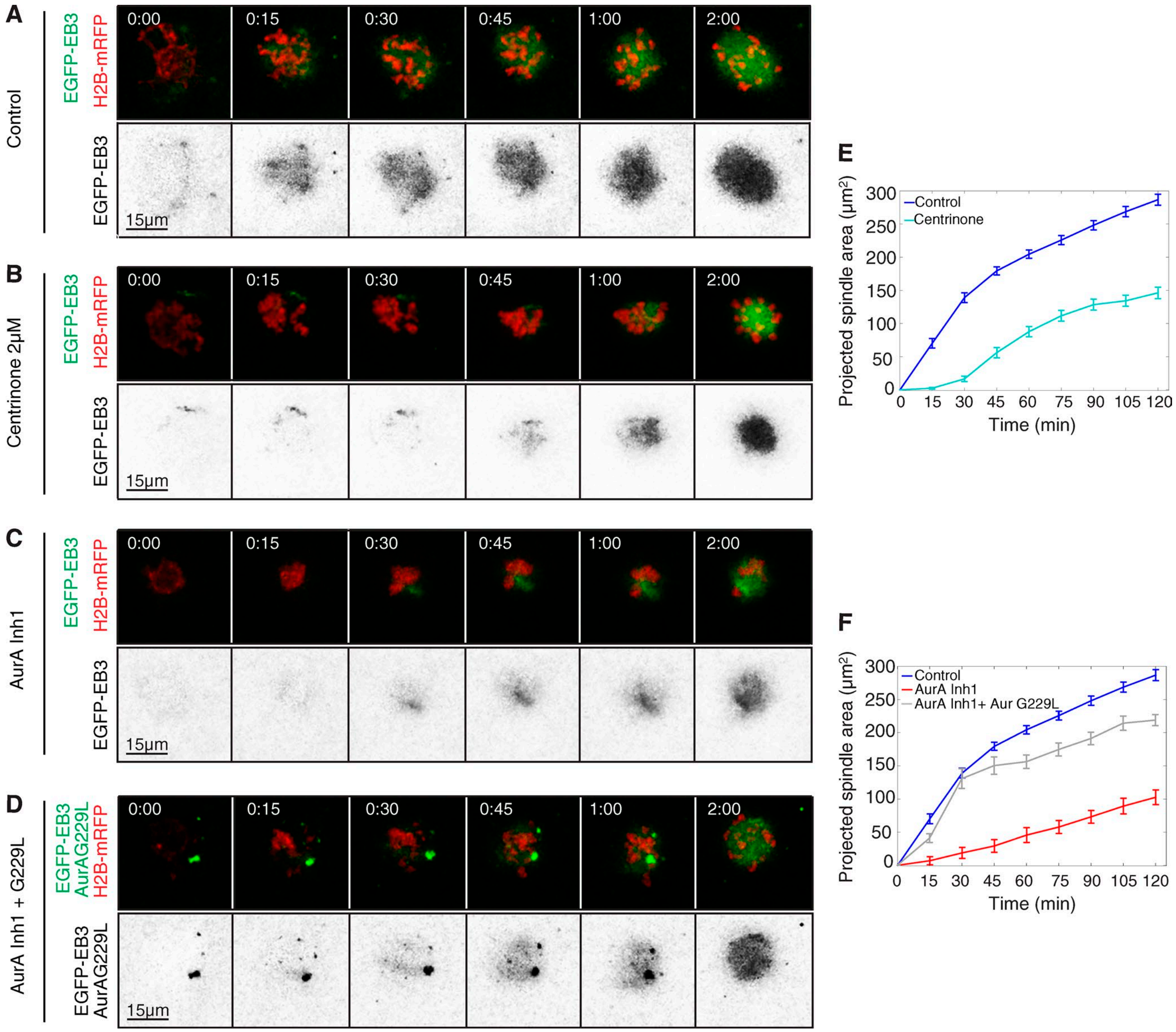

Figure S2. **Plk4 and Aurora A facilitate microtubule nucleation upon meiotic resumption.** (A–C) Time-lapse series of oocytes injected with EGFP-EB3 (black, inverted) and histone H2B-mRFP (red) mRNAs. Progression through early stages of meiosis I was imaged in untreated control conditions (A) or in the presence of 2 µM centrinone (B) and Aurora A inhibitor 1 (AurA Inh1; C). (D) To assess reversion of the Aurora A inhibition phenotype caused by Aurora A inhibitor 1, oocytes were coinjected with mRNA to express AurA Kinase G229L. Time (in hours:minutes) relative to NEBD. (E) Quantification of the size of the projected spindle area. Plk4 inhibition by centrinone at 2 µM (*n* = 23). Centrinone significantly reduces the kinetics of microtubule growth, as determined by the size of the spindle area, compared with controls (*n* = 44). Error bars indicate SEM. P-values are shown in Table S1. See also Videos 1 and 2 and Figs. 1 and 2. (F) Quantification of the size of the projected spindle area throughout time after Aurora A inhibition with Aurora A inhibitor 1 (*n* = 10). Defects caused by Aurora A inhibitor 1 treatment are significantly rescued by coexpression of Aurora A kinase G229L (*n* = 17). Error bars indicate SEM. P-values are shown in Table S1.

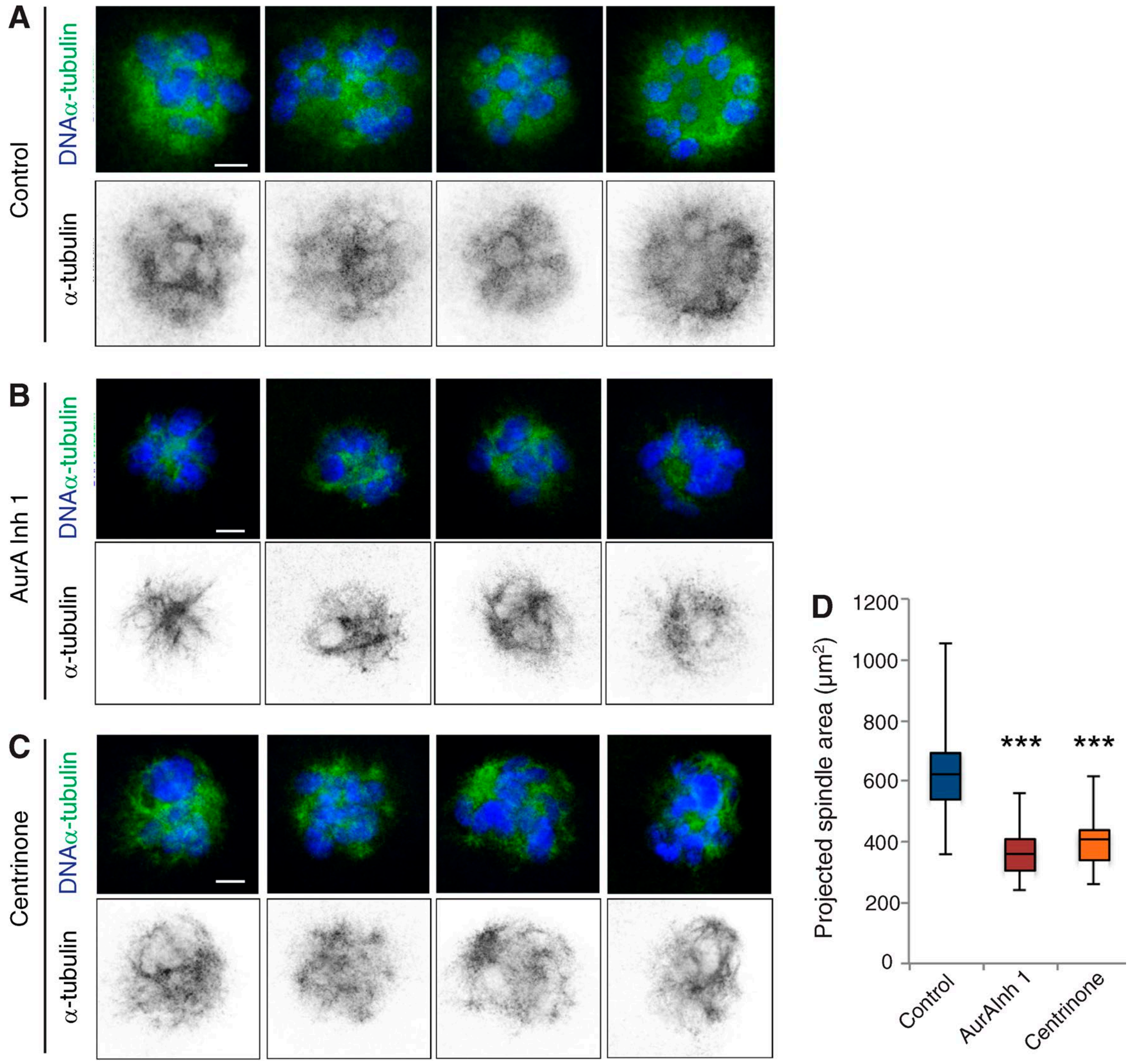

Figure S3. **Plk4 and Aurora A facilitate microtubule nucleation upon meiotic resumption.** (A–C) Immunostaining of oocytes at prometaphase (120 min after NEBD) to reveal α-tubulin (green, top; inverted, bottom) and DNA (blue, top). Depicted are oocytes that underwent meiosis in control conditions (A), in the presence of Aurora A inhibitor 1 (B), and treated with the Plk4 inhibitor centrinone (C). (D) Quantification of spindle area of oocytes fixed and stained for α-tubulin. Inhibition of Plk4 and Aurora A reduces the area of the spindle significantly. Error bars represent SD of the mean (***, $P < 0.001$). Bars, 5 μm.

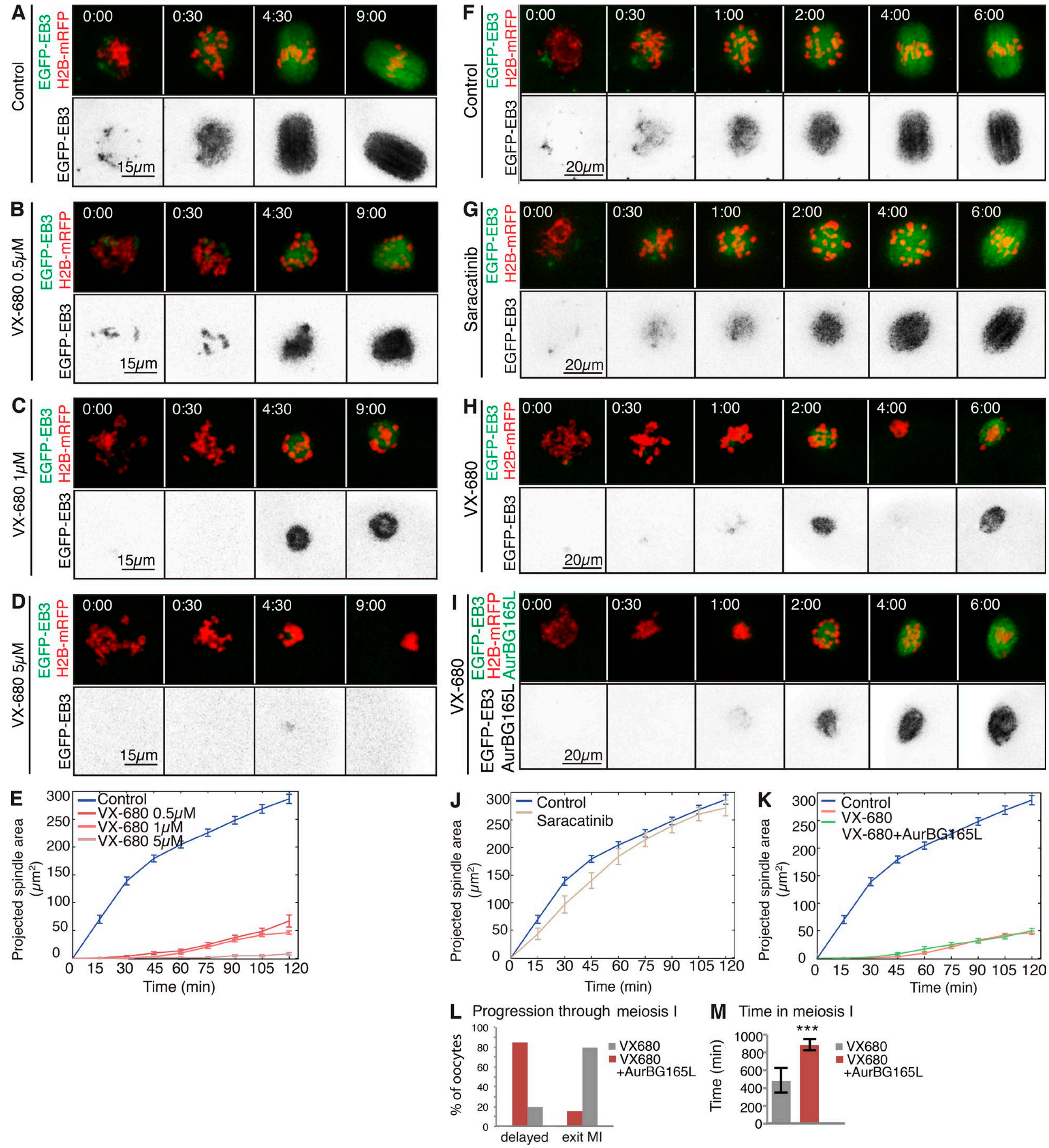

Figure S4. **VX-680–mediated inhibition of Plk4 and Aurora A diminishes microtubule nucleation.** Dose–response of oocytes to increased concentrations of VX-680. (A–C) Time-lapse series of oocytes injected with mRNA encoding EGFP-EB3 (green, top; inverted, bottom) and H2B-mRFP (red, top). Meiosis I was imaged in untreated control conditions (A) and in oocytes treated with 0.5 μM V-680 (B; *n* = 11), 1 μM VX-680 (C; *n* = 27), and 5 μM VX-680 (D; *n* = 11). (E) Size of the projected spindle area plotted over time, quantified in oocytes undergoing meiosis I in the conditions in A–D. Error bars indicate SEM. For p-values, see Table S1. (F–I) Time-lapse series of oocytes injected with EGFP-EB3 (green, top; inverted, bottom) and H2B-mRFP (red, top) mRNAs. Meiosis I was imaged in untreated control conditions (F) and in the presence of 1 μM of the Src kinase inhibitor saracatinib (G). (H and I) Time-lapse series of oocytes expressing EGFPEB3 (green, top; inverted, bottom) and H2B-mRFP (red, top) mRNA undergoing meiosis I in the presence of VX-680. Treatment with VX-680 (H) induces exit of meiosis I (7:30) and polar body extrusion failure, followed by the assembly of a meiosis II spindle (14:00). Expression of Aurora B G165L (I) is able to rescue polar body extrusion defects, and a meiosis I spindle remains until 14:00. Time (in hours:minutes) relative to NEBD. (J and K) Size of the projected spindle area measured throughout time. (J) Inhibition of Src tyrosine kinases (*n* = 12) has no effect on microtubule growth compared with controls (*n* = 44). Error bars indicate SEM. (K) Treatment of oocytes with 1 μM VX-680 (*n* = 26) significantly reduces the spindle area compared with controls (*n* = 44) but does not significantly differ in oocytes expressing Aurora B G165L (*n* = 9). Error bars represent SEM. (L) Percentage of oocytes that are delayed in meiosis I (MI) or exit meiosis I during 20 h of imaging, when followed in the presence of VX-680 with or without expression of Aurora B G165L. VX-680 treatment (*n* = 60) causes 80% of the oocytes to exit meiosis I with polar body extrusion failure (see H), which is reduced to 15% in oocytes expressing Aurora B G165L (*n* = 20). (M) Mean time spent in meiosis I in the presence of VX-680 is significantly prolonged as a result of Aurora B G165L expression (***, $P < 0.001$). Error bars indicate SD of mean.

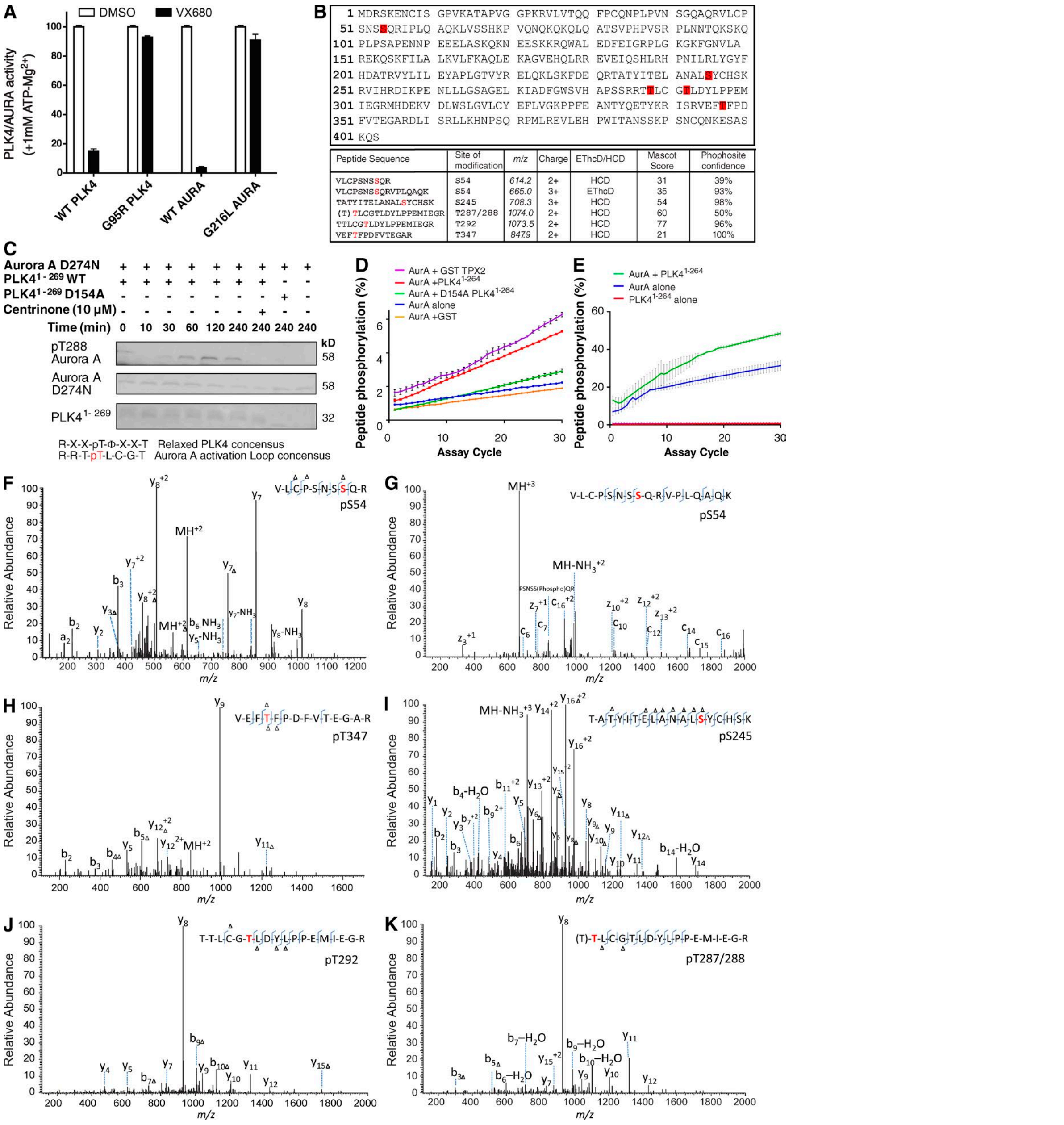

Peptide Sequence	Site of modification	m/z	Charge	EThcD/HCD	Mascot Score	Phophosite confidence
VLCPSNSSQR	S54	614.2	2+	HCD	31	39%
VLCPSNSSQRVPLQAQK	S54	665.0	3+	EThcD	35	93%
TATYITELANALSYCHSK	S245	708.3	3+	HCD	54	98%
(T)TLCGTLDYLPPEMIEGR	T287/288	1074.0	2+	HCD	60	50%
TTLCGTLDYLPPEMIEGR	T292	1073.5	2+	HCD	77	96%
VEFTFPDFVTEGAR	T347	847.9	2+	HCD	21	100%

Figure S5. **Plk4 phosphorylates Aurora A on five phosphosites in vitro.** (A) Purified recombinant WT or G95R human PLK4 (amino acids 1–284) or WT or G216L (equivalent to murine G229L) full-length human Aurora A was assayed by quantified mobility shift using EZ Reader technology with the appropriate Aurora A or PLK4 fluorescent peptide substrate in the presence of DMSO (control) or VX-680 (10 M), in the presence of 1 mM ATP (final concentration), to mimic cellular ATP levels. (B) Purified recombinant WT PLK4 was incubated with D274N Aurora A (+/– 1 mM Mg-ATP) for 2 h at 37°C before in-solution digestion and analysis by LC-MS/MS. PLK4 phosphorylates Aurora A on five sites (S54, S245, T287/T288, T292, and T347), which are highlighted in red (top). Peptide sequence with associated m/z values are depicted with Mascot scores and phosphosite confidence (bottom). There was no phosphorylation of D274N Aurora A in the absence of PLK4 (1–284) or if D274N Aurora A was incubated with 1 mM Mg-ATP. (C) Purified human kinase-dead Aurora A (D274N) was incubated alone and in the presence of WT or kinase-dead (D154A) PLK4 (1–284), or in the presence of 10 mM centrinone with 1 mM Mg-ATP. Reactions were analyzed at the indicated time-points by Western blotting using the same phosphospecific antibody used for oocyte pT288 analysis. (D) L-phosphatase–treated WT Aurora A (which has very low-level basal activity caused by the enzymatic dephosphorylation of T288 but can be "reactivated" by activating factors) was incubated with the indicated purified proteins for 30 min at 37°C in the presence of Mg-ATP, and Aurora A activity was then assayed by the inclusion of a fluorescent Aurora A substrate peptide derived from the consensus kemptide sequence. Activity was analyzed in kinetic mode for 30 cycles and converted to total peptide phosphorylation. (E) WT Aurora A, which is highly active after expression in bacteria, was assayed for hyperactivation as in D in the presence or absence of WT PLK4 (1–284). Control kinetic reactions containing either peptide or PLK4 (1–284) alone exhibited no kemptide phosphorylation. Data are presented as the mean ± SD for duplicate reactions, with similar results seen in at least one independent experiment. (F–K) MS2 spectra generated by HCD or EThcD of tryptic phosphopeptides derived from human kinase-dead (D274N) Aurora A phosphorylated by PLK4 (1–285). The peptide sequence and the identified phosphosite is detailed on each mass spectrum. (F) Doubly charged ion at m/z 614.2, generated by HCD, identifying pS54; (G) triply charged ion at m/z 665.0, generated by EThcD, providing additional evidence for pS54; (H) doubly charged ion at m/z 847.9, generated by HCD, identifying pT347; (I) triply charged ion at m/z 708.3, generated by HCD, identifying pS245; (J) doubly charged ion at m/z 1,073.5, generated by HCD identifying pT292; and (K) doubly charged ion at m/z 1,074.0, generated by HCD, identifying the activating T-loop residue pT287 or pT288. The presence of pT288 on Aurora A after incubation with PLK4 was confirmed by Western blotting using a pT288 phosphospecific antibody.

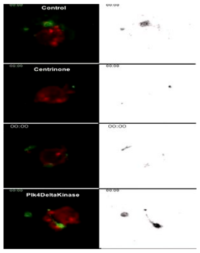

Video 1. **Plk4 is required for acentriolar spindle assembly.** Oocytes expressing EGFP-EB3 (green, right; inverted black, left) and histone H2B-mRFP (red, right). Progression through the early stages of meiosis was imaged under the indicated conditions. Treatment with centrinone or coexpression of Plk4Δkinase significantly delays microtubule growth. Inhibition of Plk4 with 5 µM centrinone can be partially rescued by expression of a drug-resistant Plk4 allele (G95R). Z stacks are composed of 20 optical sections every 3 µm. Images were acquired every 15 min. NEBD corresponds to time 00:00 (hours:minutes). See also Fig. 1 (A–E).

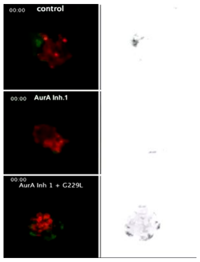

Video 2. **Aurora A plays a role in acentriolar spindle assembly.** Oocytes injected with mRNA encoding for EGFP-EB3 (gray, right; inverted black, left) and histone H2B-mRFP (red, right) to follow the dynamics of microtubule nucleation and spindle assembly. Progression through the early stages of meiotic maturation is shown. Imaging was performed in conditions as indicated (untreated control, treatment with 1 µM Aurora A inhibitor 1). Compared with controls, inhibition of Plk4 causes a significant delay in the initiation of microtubule growth that can be rescued by expression of a drug-resistant Aurora A G229L. Z stacks are composed of 20 optical sections 3 µm apart. Images were acquired every 15 min. NEBD corresponds to time 00:00 (hours:minutes). See also Fig. 2 (A and B) and Fig. S2.

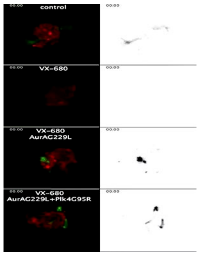

Video 3. **Aurora A predominates over Plk4 to initiate microtubule nucleation.** Time-lapse imaging of oocytes injected with mRNA encoding EGFP-EB3 (green, right; inverted black, left), and histone H2B-mRFP (red, right panels). Shown is progression through the early stages of meiosis under the indicated conditions. Treatment with VX-680 significantly delays microtubule growth, and these defects can be restored by expression of drug-resistant alleles of Aurora A (G229L) and Plk4 (G95R). Z stacks are composed of 20 optical sections every 3 µm. Images were acquired every 15 min. NEBD corresponds to time 00:00 (hours:minutes). See also Figs. 5 and S4.

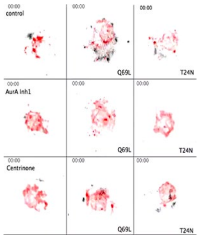

Video 4. **Ran has distinct functions from Aurora A and Plk4 during spindle assembly of meiosis I.** Time-lapse imaging of oocytes injected with mRNAs for EGFP-EB3 (inverted, black) and histone H2B-mRFP (control), dominant-negative Ran (T24N), or constitutively active Ran (Q69L). Progression through meiosis I was followed either in untreated conditions (control) or in the presence of Aurora A inhibitor 1 or centrinone. Expression of RanT24N further reduces microtubule nucleation. RanQ69L is not able to rescue the initial delay of microtubule nucleation caused by inhibition of Aurora A and Plk4; however, it is able to increase the amount of nucleated microtubules at later stages of meiosis progression. Z stacks are composed of 20 optical sections every 3 µm. Images were acquired every 15 min. NEBD corresponds to time 00:00 (hours:minutes). See also Fig. 7.

Table S1. **P-values for size of projected spindle area**

Condition	15 min	30 min	45 min	60 min	75 min	90 min	115 min	120 min
Control versus 1 μM AurA Inh 1	6.4×10^{-10}	9.5525×10^{-11}	9.557×10^{-11}	9.557×10^{-11}	9.557×10^{-11}	9.5525×10^{-11}	9.557×10^{-11}	9.557×10^{-11}
Control versus 2 μM AurA Inh 1	0.00055725	0.00029832	0.00029832	0.00029832	0.00029832	0.00029832	0.00029832	0.00029832
Control versus 5 μM AurA Inh 1	1.2339×10^{-6}	3.7412×10^{-7}	3.7431×10^{-7}	3.7431×10^{-7}	3.7431×10^{-7}	3.7431×10^{-7}	3.7431×10^{-7}	3.7431×10^{-7}
Control versus AurA Inh 1 + centrinone	3.0624×10^{-6}	0.000001019	0.000001019	0.000001019	0.000001019	0.000001019	0.000001019	0.000001019
Control versus AurA Inh 1 + RanT24N	2.6688×10^{-7}	6.9596×10^{-8}	5.6386×10^{-8}	5.6386×10^{-8}	5.6386×10^{-8}	5.6386×10^{-8}	5.6386×10^{-8}	5.6386×10^{-8}
Control versus AurA Inh 1 + RanQ69L	6.9716×10^{-7}	4.6063×10^{-7}	2.4454×10^{-7}	1.591×10^{-7}	1.974×10^{-7}	1.7725×10^{-7}	1.4275×10^{-7}	1.4275×10^{-7}
Control versus BI2536	0.012183	0.000051798	1.3604×10^{-6}	1.9255×10^{-6}	9.5662×10^{-7}	3.2343×10^{-7}	1.3948×10^{-7}	9.5251×10^{-8}
Control versus 2 μM centrinone	5.1942×10^{-10}	9.3422×10^{-11}	8.5589×10^{-11}	8.5589×10^{-11}	2.6366×10^{-10}	2.2217×10^{-10}	7.84×10^{-11}	9.3422×10^{-11}
Control versus centrinone + RanQ69L	6.8605×10^{-6}	7.2544×10^{-7}	2.1875×10^{-7}	1.975×10^{-7}	1.975×10^{-7}	1.6083×10^{-7}	8.6115×10^{-8}	1.062×10^{-7}
Control versus centrinone + RanT24N	6.9716×10^{-7}	2.1975×10^{-7}	1.4275×10^{-7}	1.4275×10^{-7}	1.4275×10^{-7}	1.4275×10^{-7}	1.4275×10^{-7}	1.4275×10^{-7}
Control versus 5 μM centrinone	3.6307×10^{-11}	4.08×10^{-12}	4.08×10^{-12}	3.7436×10^{-12}	4.4459×10^{-12}	3.7436×10^{-12}	3.7436×10^{-12}	4.08×10^{-12}
Control versus Plk4Dkinase	5.4012×10^{-7}	7.7505×10^{-8}	5.6386×10^{-8}	5.6386×10^{-8}	5.6386×10^{-8}	5.6386×10^{-8}	5.6386×10^{-8}	5.6386×10^{-8}
Control versus MLN8237	0.00055725	0.00029832	0.00029832	0.00029832	0.00029832	0.00029832	0.00029832	0.00029832
Control versus RanQ69L	0.063347	0.72869	0.19393	0.22344	0.052579	0.017939	0.0041921	0.0063853
Control versus RanT24N	4.1174×10^{-7}	8.3458×10^{-9}	1.1505×10^{-9}	9.4751×10^{-9}	1.0442×10^{-9}	8.5956×10^{-10}	8.5956×10^{-10}	8.5956×10^{-10}
Control versus saracatinib	0.067683	0.006039	0.017969	0.19776	0.4661	0.61062	0.74179	0.4661
Control versus 1 μM VX680	1.9423×10^{-11}	2.0671×10^{-12}	2.0671×10^{-12}	2.068×10^{-12}	2.068×10^{-12}	2.068×10^{-12}	2.0671×10^{-12}	2.068×10^{-12}
Control versus 0.5 μM VX680	1.3716×10^{-6}	3.7412×10^{-7}	3.7431×10^{-7}	3.7431×10^{-7}	3.7431×10^{-7}	3.7431×10^{-7}	3.7431×10^{-7}	3.7431×10^{-7}
Control versus 5 μM VX680	1.2339×10^{-6}	3.7412×10^{-7}	3.7431×10^{-7}	3.7431×10^{-7}	3.7431×10^{-7}	3.7431×10^{-7}	3.7431×10^{-7}	3.7431×10^{-7}
Control versus VX680 + AurBG165L	7.9863×10^{-6}	2.8848×10^{-6}	2.8862×10^{-6}	2.8862×10^{-6}	2.8862×10^{-6}	2.8862×10^{-6}	2.8862×10^{-6}	2.8862×10^{-6}
VX680 versus VX680 G95R	0.3758	0.0016	0.0019	0.0131	0.0766	0.3183	0.5196	0.1868
VX680 versus VX680 G229L	3.54×10^{-6}	0.0001	0.0001	0.0006	8.20×10^{-6}	1.85×10^{-7}	6.13×10^{-8}	6.13×10^{-8}
VX680 versus VX680 G229L G95R	5.89×10^{-5}	0.00043517	9.14×10^{-6}	1.32×10^{-7}	1.14×10^{-7}	1.14×10^{-7}	2.02×10^{-7}	1.52×10^{-7}
VX680 versus VX680 G229LKD	0.375871202	0.001605856	0.001998491	0.013187402	0.076647698	0.318325298	0.519675896	0.186898398
VX680 versus VX680 G229L versus VX680 G229L G95R	3.54×10^{-6}	0.000140738	0.000199965	0.000662382	8.20×10^{-6}	1.85×10^{-7}	6.13×10^{-8}	6.13×10^{-8}
5 μM centrinone versus 5 μM centrinone + G95R	5.89×10^{-5}	0.00043517	9.14×10^{-6}	1.32×10^{-7}	1.14×10^{-7}	1.14×10^{-7}	2.02×10^{-7}	1.52×10^{-7}
AurA Inh 1 versus AurA Inh 1 + RanT24N	0.606773474	0.047058898	0.017578487	0.000300665	9.69×10^{-5}	0.000129373	7.21×10^{-5}	0.000112097
AurA Inh 1 versus AurA Inh 1 + RanQ69L	0.203186877	0.231006538	0.049441661	0.149633765	0.238454775	0.238415544	0.045264604	0.01578488
Centrinone versus centrinone + RanT24N	0.74128827	0.169839443	0.005148887	0.001660344	0.001660344	0.001868721	0.009624001	0.012962541

Condition	15 min	30 min	45 min	60 min	75 min	90 min	115 min	120 min
Centrinone versus centrinone + RanQ69L	0.04453514	0.323132062	0.668540789	0.553298127	0.668540789	0.391830149	0.121638544	0.075325766
AurA Inh 1 versus AurA Inh 1 + Aurora A G229L	0.9949	0.10510	0.0001	0.0001	0.0001	0.0001	0.0001	0.0001
Control versus AurA Inh 1 + Aurora A G229L	0.85170	0.01350	0.42900	0.0137	0.0001	0.0001	0.0001	0.0001

AurA Inh 1, Aurora A inhibitor 1.

Table S2. **Values of t50 and slope together with their relative confidence intervals of the four parameters logistic function fitted on the spindle area quantifications**

Experimental conditions	t50	95% confidence interval t50		Slope	95% confidence interval slope	
Control (*n* = 44)	38.658	30.995	46.321	0.020352	0.013328	0.027376
1 µM AurA Inh 1 (*n* = 21)	55.008	52.286	57.731	0.032656	0.026754	0.038559
2 µM AurA Inh 1 (*n* = 5)	74.533	72.889	76.177	0.032978	0.02933	0.036627
5 µM AurA Inh 1(*n* = 6)	86.767	73.849	99.686	0.022378	0.0080442	0.036712
AurA Inh 1 + centrinone (*n* = 10)	72.486	69.974	74.999	0.028825	0.024541	0.033109
AurA Inh 1 + RanT24N (*n* = 13)	67.998	64.954	71.042	0.024239	0.020526	0.027951
AurA Inh 1 + RanQ69L (*n* = 12)	56.429	50.999	61.86	0.023622	0.017333	0.029911
BI2536 (*n* = 15)	41.12	33.943	48.298	0.020018	0.013683	0.026354
2 µM centrinone (*n* = 23)	55.425	51.87	58.98	0.028567	0.022641	0.034492
Centrinone + RanQ69L (*n* = 13)	57.816	54.195	61.437	0.022653	0.018776	0.02653
Centrinone + RanT24N (*n* = 12)	68.56	66.378	70.742	0.026378	0.023252	0.029503
5 µM centrinone (*n* = 26)	66.966	63.872	70.06	0.028869	0.0236	0.034139
Centrinone + Plk4G95R (*n* = 25)	47.913	43.63	52.196	0.022972	0.018217	0.027727
Plk4Δkinase (*n* = 13)	73.431	70.088	76.774	0.023294	0.019477	0.027112
MLN8237 (*n* = 10)	72.77	71.343	74.198	0.031551	0.028649	0.034453
RanQ69L (*n* = 22)	42.447	35.434	49.459	0.021722	0.014597	0.028847
RanT24N (*n* = 18)	61.174	56.454	65.895	0.019169	0.015426	0.022912
Saracatinib (*n* = 12)	45.521	41.265	49.778	0.022371	0.017847	0.026895
1 µM VX-680 (*n* = 26)	77.784	76.482	79.086	0.034691	0.031493	0.03789
0.5 µM VX-680 (*n* = 11)	84.009	79.265	88.753	0.025077	0.018689	0.031465
5 µM VX-680 (*n* = 11)	86.767	73.849	99.686	0.022378	0.0080442	0.036712
VX-680 + AurBG165L (*n* = 9)	76.275	71.776	80.775	0.023603	0.018307	0.028899
VX-680 + AurAG229L (*n* = 15)	62.845	60.241	65.448	0.027088	0.023176	0.031001
VX-680 + AurAG229LKD (*n* = 8)	92.308	88.018	96.599	0.032611	0.022939	0.042284
VX-680 + AurAG229L + Plk4G95R (*n* = 14)	47.862	45.932	49.792	0.02801	0.024898	0.031122
VX-680 + Plk4G95R (*n* = 10)	66.39	62.393	70.387	0.022694	0.018389	0.026999
AurA Inh 1 + AurAG229L (*n* = 17)	26.97	21.86	33.5	0.02929	0.01985	0.04647

Global kinetics was monitored over a 120-min time window. Fitting is based on all available independent measurements (*n*). AurA Inh 1, Aurora A inhibitor 1.

Table S3. **Inhibitor concentrations**

Compound	Concentration	Remarks
BI2536	100 nM	Concentration maximally reduces phosphorylation of BubR1 at T669 in mouse oocytes (Solc et al., 2015); and abolishes Plk1 kinase activity in vitro (Scutt et al., 2009)
MLN8237	500 nM	Efficiently prevents Aurora A activation and activity in vivo (Hochegger et al., 2013)
Aurora A inhibitor 1	1–5 µM	Concentration suppresses mitotic Aurora A autophosphorylation on Thr288 in vitro (Yuan et al., 2012)
Aurora A inhibitor 1	Dose–response	Increasing concentrations above 1 µM to 2 µM or 5 µM does not significantly further affect microtubule growth (not depicted)
Centrinone	2–5 µM	1–10 µM inhibits centriole assembly in Xenopus embryos (Wong et al., 2015); 5 µM phenocopies effect of Plk4ΔKinase in the mouse oocyte (Fig. 1; compare Fig. S1); treatment of 2-cell embryos with 2 µM phenocopies Plk4dsRNAi (Fig. S1; compare Coelho et al., 2013); phenotype induced by 5 µM centrinone is reversible by expression of Plk4 G95R (Fig. 1, C and E)
VX-680	0.5–5 µM	1 µM abolishes Aurora A kinase activity in vitro but not that of inhibitor-resistant (G216L) Aurora A (Scutt et al., 2009)
VX-680	Dose–response (Fig. S4)	Phenotype caused by treatment with VX-680 at 1 µM concentration is reversible by expression of inhibitor-resistant (G216L equivalent) Aurora A (Fig. 5)
Saracatinib	1 µM	Used at 1 µM to inhibit Src kinases in vivo (Liu et al., 2011)

Table S4. **FRAP values for $t_{1/2}$, mobile fraction, and their relative confidence intervals**

Condition	$t_{1/2}$	95% confidence interval $t_{1/2}$		Mobile fraction	95% confidence mobile fraction	
**Cytoplasmic MTOCs, stage 1**						
Control (n = 10)	2.193	2.085	2.306	0.6092	0.6028	0.6158
Centrinone (n = 12)	2.988	2.887	3.092	0.6616	0.6556	0.6678
AurA Inh 1 (n = 10)	2.300	2.045	2.582	0.5010	0.4886	0.5142
RAN (n = 10)	1.938	1.842	2.038	0.6349	0.6284	0.6415
**Cytoplasmic MTOCs, stage 2**						
Control (n = 14)	1.930	1.813	2.053	0.5827	0.5752	0.5903
Centrinone (n = 15)	2.414	2.338	2.493	0.6526	0.6478	0.6576
AurA Inh 1 (n = 15)	2.267	2.120	2.422	0.6375	0.6284	0.6469
RAN (n = 14)	3.700	3.492	3.922	0.6464	0.6348	0.6586
**Cytoplasmic MTOCs, stage 3**						
Control (n = 14)	2.221	2.106	2.341	0.6482	0.6409	0.6558
Centrinone (n = 13)	2.431	2.350	2.514	0.6390	0.6341	0.6440
AurA Inh 1 (n = 9)	2.275	2.155	2.400	0.6030	0.5960	0.6103
RAN (n = 9)	2.761	2.626	2.901	0.6327	0.6249	0.6409
**MTOCs at chromosomes, stage 1**						
Control (n = 7)	1.745	1.621	1.875	0.6371	0.6286	0.6458
Centrinone (n = 13)	2.397	2.270	2.529	0.6141	0.6065	0.6219
AurA Inh 1 (n = 19)	2.666	2.474	2.870	0.6080	0.5968	0.6198
RAN (n = 12)	2.849	2.695	3.012	0.6455	0.6365	0.6549
**MTOCs at chromosomes, stage 2**						
Control (n = 10)	2.165	2.049	2.286	0.4968	0.4912	0.5026
Centrinone (n = 12)	2.397	2.227	2.578	0.6556	0.6450	0.6666
AurA Inh 1 (n = 10)	2.732	2.500	2.983	0.5706	0.5583	0.5836
RAN (n = 10)	3.253	3.081	3.436	0.6580	0.6685	0.6810
**MTOCs at chromosomes, stage 3**						
Control (n = 10)	1.896	1.763	2.036	0.5693	0.5609	0.5778
Centrinone (n = 12)	2.528	2.377	2.686	0.6660	0.6567	0.6757
AurA Inh 1 (n = 10)	2.078	1.915	2.250	0.6129	0.6026	0.6235
RAN (n = 10)	3.076	2.842	3.329	0.5749	0.5626	0.5879

AurA Inh 1, Aurora A inhibitor 1.

Table S5. **Sequences of oligonucleotides**

Oligonucleotide	Sequence (5′ to 3′)
RanQ69L_Fwd	AGTTCAATGTATGGGACACAGCCGGCCTGGAGAAATTCGGTGGACTGAGAGATGG
RanQ69L_Rev	CCATCTCTCAGTCCACCGAATTTCTCCAGGCCGGCTGTGTCCCATACATTGAACT
AurAG229L_Fwd	CTGATTCTAGAATATGCGCCCCTTCTAACAGTCTATAGAGAGCTCC
AurAG229L_Rev	GGAGCTCTCTATAGACTGTTAGAAGGGGCGCATATTCTAGAATCAG
Plk4G95R_Fwd	CTGGTATTAGAAATGTGCCATAATAGAGAAATGAACAGGTATCTAAAGAATAG
Plk4G95R_Rev	CTATTCTTTAGATACCTGTTCATTTCTCTATTATGGCACATTTCTAATACCAG
AurBG165L_Fwd	CCTGGAATACGCCCCTCGCCTGGAACTCTACAAGGAACTG
AurBG165L_Rev	CAGTTCCTTGTAGAGTTCCAGGCGAGGGGCGTATTCCAGG
AurAK175R_Fwd	GCAAGTTCATCCTGGCTCTGAGGGTGCTGTTTAAAACACAGCTGG
AurAK175R_Rev	CCAGCTGTGTTTTAAACAGCACCCTCAGAGCCAGGATGAACTTGC

Fwd, forward; Rev, reverse.

References

Coelho, P.A., L. Bury, B. Sharif, M.G. Riparbelli, J. Fu, G. Callaini, D.M. Glover, and M. Zernicka-Goetz. 2013. Spindle formation in the mouse embryo requires Plk4 in the absence of centrioles. *Dev. Cell.* 27:586–597. https://doi.org/10.1016/j.devcel.2013.09.029

Hochegger, H., N. Hégarat, and J.B. Pereira-Leal. 2013. Aurora at the pole and equator: overlapping functions of Aurora kinases in the mitotic spindle. *Open Biol.* 3:120185. https://doi.org/10.1098/rsob.120185

Liu, H.S., C.S. Ke, H.C. Cheng, C.Y.F. Huang, and C.L. Su. 2011. Curcumin-induced mitotic spindle defect and cell cycle arrest in human bladder cancer cells occurs partly through inhibition of aurora A. *Mol. Pharmacol.* 80:638–646. https://doi.org/10.1124/mol.111.072512

Scutt, P.J., M.L. Chu, D.A. Sloane, M. Cherry, C.R. Bignell, D.H. Williams, and P.A. Eyers. 2009. Discovery and exploitation of inhibitor-resistant aurora and polo kinase mutants for the analysis of mitotic networks. *J. Biol. Chem.* 284:15880–15893. https://doi.org/10.1074/jbc.M109.005694

Solc, P., T.S. Kitajima, S. Yoshida, A. Brzakova, M. Kaido, V. Baran, A. Mayer, P. Samalova, J. Motlik, and J. Ellenberg. 2015. Multiple requirements of PLK1 during mouse oocyte maturation. *PLoS One.* 10:e0116783. https://doi.org/10.1371/journal.pone.0116783

Wong, Y.L., J.V. Anzola, R.L. Davis, M. Yoon, A. Motamedi, A. Kroll, C.P. Seo, J.E. Hsia, S.K. Kim, J.W. Mitchell, et al. 2015. Cell biology. Reversible centriole depletion with an inhibitor of Polo-like kinase 4. *Science.* 348:1155–1160. https://doi.org/10.1126/science.aaa5111

Yuan, H., Z. Wang, H. Zhang, M. Roth, R. Bhatia, and W.Y. Chen. 2012. Overcoming CML acquired resistance by specific inhibition of Aurora A kinase in the KCL-22 cell model. *Carcinogenesis*. 33:285–293. https://doi.org/10.1093/carcin/bgr278
